# Development of nucleic acid testing technology for quarantine pest *Globodera rostochiensis* based on a species-specific DNA fragment

**DOI:** 10.7717/peerj.21155

**Published:** 2026-05-15

**Authors:** Fengqi Lan, Honghao Li, Zhenji Yin, Yajie Wang, Xiaofeng Liu, Jiahui Yang, Yue Sun, Yingqing Tang, Bingjie Wang, Shiyang Yin, Bing Li, Xiang Tao

**Affiliations:** 1 College of Life Sciences, Sichuan Normal University, Chengdu, Sichuan, China; 2 Institute of Plant Protection, Sichuan Academy of Agricultural Sciences, Chengdu, Sichuan, China; 3 Institute of Crops, Sichuan Academy of Agricultural Sciences, Chengdu, Sichuan, China

**Keywords:** Potato, *Globodera rostochiensis*, PCR, RAA-LFD, Molecular testing

## Abstract

**Background:**

*Globodera rostochiensis*, a potato cyst nematode (PCN) species, is distributed in potato-growing regions across Europe, Asia, Africa, Oceania, and the Americas. Recognized as a quarantine pest under international phytosanitary frameworks, its detection is critical for biosecurity.

**Methods:**

This study aimed to identify a *G. rostochiensis*-specific DNA fragment using comparative genomics and develop both a polymerase chain reaction (PCR)-based detection assay and a recombinase-aided amplification (RAA)-based isothermal detection assay.

**Results:**

A 3,572 bp species-specific DNA fragment was identified. Based on this marker, a conventional PCR diagnostic assay and a recombinase-aided amplification coupled with a lateral flow dipstick (RAA-LFD) diagnostic assay were successfully developed. The PCR primer pair GroF1/GroR1 exhibited a detection limit of 200 copies and a sensitivity of 10 copies/μL (5.6 × 10^−2^ fg/μL) when amplified over 32 cycles within a 20 μL PCR reaction volume. Three independent experiments demonstrated that DNA from a single larva could be stably detected using the PCR assay. Following reactions with a 25 μL RAA volume and temperature of 37.5 °C for 10, 15, 20, 25, and 30 min, the detection limit progressively decreased from 5.75 × 10^4^ to 5.75 × 10^0^ copies. The corresponding sensitivities ranged from 2.3 × 10^3^ copies/μL to 2.3 × 10^−1^ copies/μL. Across independent experiments, the RAA assay consistently detected DNA equivalent to 10^−2^ of a single larva. The RAA-LFD assay achieved 100% diagnostic sensitivity for the detection of *G. rostochiensis* in infested field soil samples following a 25 min isothermal amplification at 37.5°C. The established RAA-LFD molecular diagnostic assay exhibits marked potential for the field-deployable detection of *G. rostochiensis* in root zone soil samples, offering rapid turnaround and simplified procedural requirements.

## Introduction

Potato (*Solanum tuberosum*) ranks as the fourth most cultivated crop globally after maize, rice, and wheat (Zhang et al., 2017). Functioning as a multipurpose crop for food, vegetable, feed, and industrial feedstocks, it considerably contributes to dietary diversification, food security reinforcement, and poverty alleviation through income generation. China is the largest potato producer in the world, accounting for 24.35% of global cultivation area and 20.33% of total production in 2022 (https://www.fao.org/home/en/). *Globodera rostochiensis* is a regulated pest within the potato cyst nematodes complex, classified within the phylum Nematoda, order Tylenchida, and family Heteroderidae (genus *Globodera*). It has caused damage in more than 70 countries, and more than 100 countries and regions have listed it as a quarantine pest (Onditi & Whitworth, 2025). *G. rostochiensis* has previously appeared in southwestern China (Jiang et al., 2023), threatening multiple seed potato bases with its spread and resulting in up to 80%–90% yield loss. Currently, the detection of *G. rostochiensis* primarily relies on two methodologies: the widely accepted morphological identification and the highly accurate molecular detection. Morphological identification is based on the morphological characteristics and morphometric analyses of the sample, and requires the presence of a sufficient quantity of characteristic cysts or nematode specimens. However, the small size and subtle distinguishing characteristics of *G. rostochiensis* limit accurate observations and identification-whether by visual inspection or instrumentation. Furthermore, the instability of numerous nematode phenotypic traits complicates the acquisition of reliable morphometric data. Consequently, morphological identification is time-consuming, labor-intensive, and prone to error (Subbotin et al., 2010; Viney & Diaz, 2012; Bogale, Baniya & Di Gennaro, 2020; Wainer & Dinh, 2021; Perry & Wright, 1998) To enhance diagnostic efficiency and accuracy, molecular techniques have been widely developed and applied. Conventional polymerase chain reaction (PCR) and polymerase chain reaction-restriction fragment length polymorphism (RFLP) assays target specific genomic regions to differentiate *G. rostochiensis* from closely related species, providing significantly improved specificity and faster turnaround compared with traditional morphological diagnostics. Real-time quantitative PCR (qPCR) further enables precise quantification of target DNA and, owing to its high sensitivity and specificity, has been incorporated into official diagnostic standards such as those outlined in European and Mediterranean Plant Protection Organization (EPPO) PM 7/40 (Thiery & Mugniery, 1996; Bulman & Marshall, 1997; Mwangi et al., 2015; White et al., 1990; Kralik & Ricchi, 2017; EPPO Standard on Diagnostics, 2022). Consequently, qPCR is widely used for regulatory surveillance and confirmatory testing under controlled laboratory conditions. Although PCR-based assays perform well, they generally rely on thermal cyclers, fluorescence detection instruments, and well-equipped laboratory facilities, this dependence constrains their applicability for rapid on-site screening and poseschallenges for deployment in resource-limited settings. In addition, the substantial cost of instruments and reagents required for real-time PCR restricts its adoption in rapid, field-portable applications. In recent years, isothermal amplification methods have emerged as promising molecular alternatives that eliminate the need for thermal cycling. Among these, loop-mediated isothermal amplification (LAMP) can amplify target DNA rapidly under constant temperature and has been widely used to detecting diverse plant pathogens (Gomez-Gutierrez & Goodwin, 2022). Recombinase polymerase amplification coupled with lateral flow detection (RPA-LFD/RPA-LFA) advances this approach further, enabling rapid visual readout at low constant temperatures through integration with lateral flow strips (Dai et al., 2019; Salazar et al., 2021). Thus, the development of highly specific and sensitive molecular detection methods for *G. rostochiensis* is crucial for the effective management of this pest.

Recombinase polymerase amplification (RPA) is an isothermal nucleic acid amplification technology. It initiates with the binding of recombinase proteins to primers, forming a protein-DNA complex that recognizes and invades target double-stranded DNA (dsDNA). Single-strand DNA-binding proteins then stabilize the single-strand template. The DNA polymerase utilizes the primer to synthesize a complementary strand, enabling the exponential amplification of the target DNA (Lobato & O’Sullivan, 2018). RPA technology offers high sensitivity, strong specificity, simple operation, and low equipment requirements. Amplification can be completed within 5 to 20 min at temperatures ranging from 30 °C to 42 °C. Furthermore, the amplification products can be rapidly and visually detected using lateral flow dipsticks (LFD). Recombinase-aided amplification (RAA), a Chinese-developed technology, shares similarities with RPA but differs in its recombinase origin (Chen et al., 2018). Specifically, RAA utilizes the *Escherichia coli*-derived recombinase UvsX, while RPA employs the T4 bacteriophage-derived recombinase T4 uvsX (Piepenburg et al., 2006). Both RPA and RAA technologies have been widely applied for the rapid detection of pathogenic microorganisms. For example, Liu et al. (2023) developed an RAA assay to detect *Chlamydia felis*. Goraya & Yan (2024) established a real-time RPA assay capable of directly identifying *Paratrichodorus allius* in soil DNA extracts using portable instrumentation, achieving a sensitivity equivalent to 1/16 of a single nematode’s DNA. Cao et al. (2025) developed a one-tube dual reverse transcription RAA (RT-RAA) assay coupled with lateral flow strip detection for the rapid, on-site identification of the pepper mild mottle virus and four *Colletotrichum* species. The assay demonstrated high sensitivity with a detection limit of 0.32 copies/µL.

However, the application of RAA-LFD for detecting *G. rostochiensis* remains unreported. This study aims to integrate bioinformatics and molecular biology approaches to identify genome-specific DNA sequences of *G. rostochiensis*. These sequences can serve as molecular targets for establishing a novel recombinase-mediated isothermal nucleic acid amplification system.

## Materials & Methods

### Nematode samples

A total of 15 soil samples containing *G. rostochiensis* were analyzed. The samples were collected from experimental fields in Jing’an Town, Zhaoyang District, Zhaotong City, and cysts of *G. rostochiensis* were isolated from the soils prior to DNA extraction. Genomic DNA from additional non-target nematode species, including *Globodera mexicana*, *Heterodera glycines*, *Bursaphelenchus xylophilus*, *Aphelenchoides besseyi*, *Ditylenchus destructor*, and *Heterodera avenae*, was kindly provided by Professor Xuan Wang from the College of Plant Protection, Nanjing Agricultural University (Jiangsu Province, China). DNA of *Globodera pallida*, *Heterorhabditis indica*, and *Oscheius myriophilus* was generously supplied by Dr Shuocheng Zeng from Institute of Plant Protection, Sichuan Academy of Agricultural Sciences (Sichuan Province, China). All specimens were initially identified by experienced nematologists based on morphological characteristics, and species identity was subsequently confirmed by comparing Internal Transcript Spacer (ITS) sequences with reference entries available in GenBank.

### DNA extraction

Nematode DNA was extracted according to the protocol described by Jiang et al. (2023). Individual cysts or juveniles were transferred into 25 µL of double-distilled water in an Eppendorf tube and crushed using a pipette tip. Subsequently, three µL of 10× PCR buffer (AP111-11; TransGen Biotech Co., Ltd, Beijing, China) and 2 µL of proteinase K (20 mg/mL, GE201-01, TransGen Biotech Co., Ltd) were added, followed by incubation at 65 °C for 1 h and then at 95 °C for 10 min. After incubation, the sample was centrifuged at 12,000 rpm for 1 min. The DNA-containing supernatant was then transferred to a new Eppendorf tube and stored at −20 °C until further analysis.

### Comparative genomics analysis

The executable BLAST+ software (v2.4.0) was obtained from the NCBI FTP site (https://ftp.ncbi.nlm.nih.gov/blast/executables/blast+/LATEST/) and installed on a tower server to establish a local BLAST platform. The genomes of *G. rostochiensis* (GCA_018350325.1, GCA_038996415.1, GCA_018350315.1, and GCA_900079975.1) and its closely related species (*G. pallida*: GCA_020449905.1, GCA_023343765.1, GCA_000724045.1; and *Globodera ellingtonae*: GCA_001723225.1) were retrieved from the NCBI genome database (https://www.ncbi.nlm.nih.gov/datasets/genome/?taxon=31242) and formatted by the local BLAST platform. All publicly available high-throughput sequencing reads for *G. rostochiensis* (up to May 10, 2023) were subsequently downloaded from NCBI’s Sequence Read Archive (SRA, https://www.ncbi.nlm.nih.gov/sra). These reads were then aligned against the reference genomes of *G. ellingtonae* and *G. pallida*. Reads failing to align to either genome were extracted and realigned against the *G. rostochiensis* genome. Successfully aligned reads were retained and assembled to generate candidate species-specific sequences. The Nucleotide Sequence Database (NT database) was downloaded from the NCBI FTP site (https://ftp.ncbi.nlm.nih.gov/blast/db/), and *G. rostochiensis-* related nucleic acid sequences were deleted. The candidate sequences were compared with the NT database, and successfully aligned sequences were removed. The Guanine-Cytosine (GC) content of the remaining candidate sequences was then calculated. Sequences with abnormal GC content were deleted, and the remaining sequences were used as species-specific sequences.

### Design and selection of PCR primers

*G. rostochiensis* cysts were isolated using a modified flotation technique. Cysts morphologically consistent with those reported in reference (Bellvert, Crombie & Horgan, 2008) were selected for further analysis. Genomic DNA was extracted from these cysts and subjected to electrophoresis according to the method described by Ou et al. (2008) and Baklawa et al. (2015). Species identification was performed using the primers ITS5/PITSr3 (Table 1), as specified in the Industry Standard of the People’s Republic of China for Entry-Exit Inspection and Quarantine (SN/T 1723.1-2022) (Song et al., 2022). PCR primers were designed with Primer Premier 5.0 (PREMIER Biosoft, San Francisco, California, USA) targeting species-specific genomic DNA fragments of *G. rostochiensis*. PCR amplification was performed in 20 μL reactions using the 2 ×EasyTaq^®^ PCR SuperMix (AS111-11; TransGen Biotech Co., Ltd) according to the manufacturer’s protocol. Each reaction contained: 10 μL of 2 ×EasyTaq^®^ PCR SuperMix (+dye); 1 μL of forward and reverse primers (10 μM); 1 μL genomic DNA template; and 7 μL ddH_2_O. The following thermal cycling conditions were implemented: initial denaturation at 94 °C for 3 min; 35 cycles of 94 °C for 30 s, 59 °C for 30 s, and 72 °C for 60 s; final extension at 72 °C for 5 min. Primers yielding the target amplicon under these conditions were selected for subsequent analysis.

**Table 1:** Primers and probe designed for detection of *G. rostochiensis*.

Method	Name	Sequence (5′-3′)	Product length (bp)
PCR	GroF1	GCTCTTCTTTTTACCCCACCG	1,138
	GroR1	GGCTAAGCACGAAATCCCCA	
RAA	RAA-GroF1	ATTTGTTTCCGATTTGATTCTGACCCGAGC	199
	RAA-GroR1	Bio-ATTGAGCAAAAAAGGTTCTCTTGACTATGA	
	Gro-Probe	FAM-CCAGATGTCATCACCGATATCACATTG CAC-(THF)-TTGTATTCCCATAAT-(SpacerC3)	
PCR	ITS5	GGAAGTAAAAGTCGTAACAAGG	434 (Goraya & Yan, 2024)
	PITSr3	AGCGCAGACATGCCGCAA	

### Construction of recombinant plasmids *via* Thymine-Adenine cloning

The PCR amplicons were separated *via* agarose gel electrophoresis and subsequently purified using the EasyPure Quick Gel Extraction Kit (EG101-02; TransGen Biotech Co., Ltd). The purified DNA fragments were then cloned into the pEASY-T1 Simple Cloning vector (CT111; TransGen Biotech Co., Ltd) using the Thymine-Adenine (TA) cloning method, generating recombinant plasmids harboring the target insert. The recombinant plasmids were extracted using the EasyPure^®^ Plasmid MiniPrep Kit (EM101; TransGen Biotech Co., Ltd). The concentration of the extracted plasmids was determined with an ultramicro nucleic acid-protein analyzer (ScanDrop 100; Analytik Jena, Jena, Germany) and converted to copy number. Serial tenfold dilutions, spanning from 2 × 10^−1^ to 2 × 10^6^ copies/μL, were prepared to generate a standard curve of known plasmid copy numbers.

### Establishment of a PCR detection system

With the tenfold serial diluted plasmids employed as templates, PCR amplifications were performed in 20 μL reaction volumes using the 2 ×EasyTaq^®^ PCR SuperMix (AS111-11; TransGen Biotech Co., Ltd) following the manufacturer’s protocol. The thermal cycling conditions were set as follows: initial denaturation at 94 °C for 3 min; 30 cycles of denaturation at 94 °C for 30 s, annealing at 59  °C for 30 s, and extension at 72 °C for 60 s; final extension at 72 °C for 5 min. Each reaction was performed in triplicates. The amplification products were analyzed using agarose gel electrophoresis, and plasmid dilution yielding clear and single bands of the expected size were selected for downstream applications (Mazlan et al., 2024).

To optimize the PCR conditions (Lorenz, 2012), eight 20 μL PCR reaction mixtures were prepared using the 2 ×EasyTaq^®^ PCR Super-Mix (AS111-11; TransGen Biotech Co., Ltd, China), following the manufacturer’s protocol. Reactions were run on a gradient PCR instrument with eight different annealing temperatures to determine the optimal annealing temperature (Rychlik, Spencer & Rhoads, 1990). The primer concentration was optimized by preparing five reactions with final primer concentrations ranging from 0.1 to 0.5 μM, using the previously determined optimal annealing temperature for amplification. Cycle number optimization was performed using 30–35 cycles under the optimized conditions. The detection limit was established by amplifying serial 10-fold dilutions of the recombinant plasmid using the finalized protocol. All reactions were performed in triplicate. The amplification products were analyzed *via* 1% agarose gel electrophoresis to evaluate amplicon specificity and yield.

### Establishment of the RAA-LFD detection system

The RAA isothermal amplification primers and probe were designed using Primer Premier 5.0, with the PCR-amplified fragments as the molecular target. The amplification reaction was established using the RAA-nfo nucleic acid amplification kit (S005ZC; Hangzhou ZC Bioscience, China), according to the manufacturer’s protocol. The reaction mixture consisted of: one vial of freeze-dried enzyme powder; 12.5 μL Buffer A; 1 μL each of forward and reverse primers (2 μM); 0.3 μL of probe (2 μM); 2.5 μL of recombinant plasmid DNA template; 2.5 μL Buffer B (0.5×); and ddH_2_O to a final volume of 25 μL. The reaction tubes were incubated at 39  °C for 15 min, followed by enzyme inactivation at 69 °C for 20 min to assess primer-probe efficacy. The amplification temperature and time were then optimized. Using recombinant plasmid template (2.3 × 10^9^ copies/µL), RAA amplifications were performed at nine temperatures (0.5 °C intervals across 35–39  °C) for 30 min. At the determined amplification temperature, seven different amplification times (5–35 min at 5 min intervals) were tested using the same template concentration to establish the optimal duration. Tenfold serial dilution of the recombinant plasmid was amplified under optimized conditions to determine the detection limit. All amplification products were analyzed by LFD test strips and 2% agarose gel electrophoresis. Each experiment included three technical replicates.

### Detection of *G. rostochiensis* in field-collected soil samples

Fifteen field soil samples were collected from *G. rostochiensis*-infested potato fields in 2023. Genomic DNA was extracted from each sample using the Soil DNA Extraction Kit (DPP336-02; TIANGEN Biotech, Beijing, China). DNA quality was assessed using 1% agarose gel electrophoresis. The presence of *G. rostochiensis* in the soil samples was subsequently detected using the PCR assay and the RAA-LFD system, following previously described methodologies.

## Results

### Identification of *G. rostochiensis*-specific DNA fragments

High-throughput sequencing reads of *G. rostochiensis* from the NCBI Sequence Read Archive (SRA; https://www.ncbi.nlm.nih.gov/sra) were compared with the genomes of *G. rostochiensis*, *G. pallida,* and *G. ellingtonae*. The analysis identified 407 species-specific DNA fragments for *G. rostochiensis*. Sequences characterized by a high oligonucleotide content, a length of less than 1,000 bp, and thus presenting challenges for the design of high-quality primers, were excluded. A 35,72 bp fragment was selected for further validation and subjected to NCBI BlastN analysis against the NT nucleotide database under three distinct similarity modes: “highly similar sequences”; “optimize for more dissimilar sequences”; and “optimize for somewhat similar sequences”. No significant matches were detected in these modes. The 3,572 bp fragment was located in the 9,868–13,439 region on scaffold JAEVLN010000009.1 of GCA_018350325.1. Open Reading Frame prediction confirmed the absence of a protein-coding region within this fragment. PCR primers GroF1 and GroR1 were designed based on this sequence to establish a PCR-based detection assay (Table 1).

### Development of a PCR-based detection assay

**Figure 1:**
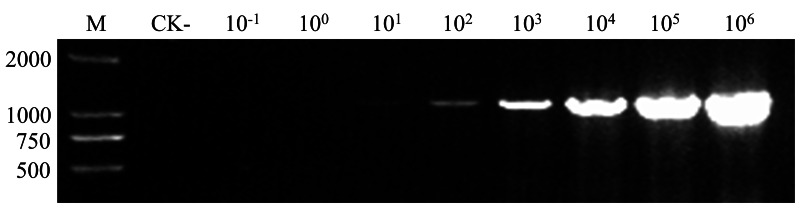
Optimization of the template concentration used in the PCR optimization process. M, DNA marker; CK-, PCR product amplified with ddH_2_O as a negative control template; Lane 3–10, PCR products amplified from the tenfold serial diluted plasmids, ranging from 2 × 10^−1^ copies/μL to 2 × 10^6^ copies/μL.

*G. rostochiensis* cysts were isolated from soil samples collected in infested areas. Genomic DNA was extracted, and species identity was confirmed as *G. rostochiensis* using the detection method specified in the Industry Standard of the People’s Republic of China for Entry-Exit Inspection and Quarantine (SN/T 1723.1-2022, employing primers ITS5 and PITSr3, Table 1), validating its suitability for subsequent experiments. PCR amplification with primers GroF1/GroR1 yielded distinct target-sized bands with no non-specific amplification. The recombinant plasmid pEASY-1138bp, containing the GroF1/GroR1 amplificon, was constructed *via* TA cloning. The concentration of the resulting plasmid was 63.98 ng/μL (7.08 × 10^9^ copies/μL). A gradient dilution series of this plasmid was subsequently prepared (2 × 10^−2^ copies/μL to 2 × 10^9^ copies/μL). Thirty-cycle PCR amplification using diluted plasmid templates showed electrophoretic band intensities proportional to DNA concentration (Fig. 1). Detectable amplification products initially appeared at 2 × 10^1^ copies/μL, with evident visible bands at 2 × 10^4^ copies/μL (Fig. 1). Consequently, 2 × 10^4^ copies/μL was selected for the subsequent annealing temperature optimization.

**Figure 2:**
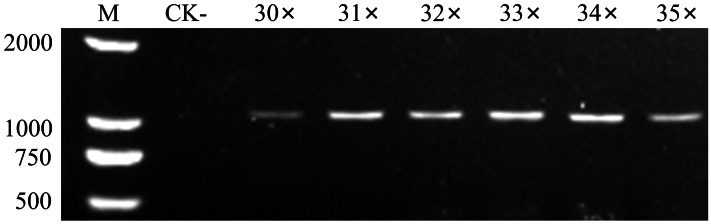
Optimization of the cycle number for the PCR amplification reaction. M, DNA marker; CK-, PCR product amplified with ddH_2_O as a negative control template; Lane 3–8, PCR products amplified with 30–35 cycles.

Primers GroF1/GroR1 successfully amplified target fragments across an annealing temperature gradient (57–62  °C), with approximately uniform electrophoretic band intensity observed throughout this range (Fig. S1). Based on the Tm values calculated using DNAMAN 5.0 (Lynnon Biosoft, San Ramon, CA, USA), 59  °C was selected as the optimal annealing temperature. To minimize the dimerization and non-specific amplification associated with high primer concentrations (Masnaini et al., 2023), a lower primer concentration may be preferable when electrophoretic band intensities are comparable. Variations in primer concentration (0.1–0.5 μM final concentration) under the 20 μL PCR reactions reveal pronounced differences in amplification efficiency, namely, a minimal band intensity at 0.1 μM, a maximal at 0.2 μM, and a plateau at 0.3–0.5 μM. Given that target DNA concentrations in diagnostic samples are typically low, 0.5 μM was selected as the working concentration to maximize sensitivity. Increasing the cycle number enhances the amplification efficiency and detection sensitivity (Budowle, Eisenberg & Van Daal, 2009). For GroF1/GroR1, the electrophoretic band brightness plateaued between 31 and 35 cycles, with no significant variation (Fig. 2), indicating that the reaction reached saturation by 30 cycles. Thus, 32 was selected as the optimal number of PCR cycles. In summary, the optimal PCR reaction conditions for the GroF1/GroR1 primers obtained by series optimization were as follows: initial denaturation at 94  °C for 3 min; 32 cycles of 94 °C for 30 s, 59 °C for 30 s, and 72 °C for 60 s; final extension at 72 °C for 5 min. Moreover, the primer concentrations in the 20 μL reaction were set as 0.5 μM.

**Figure 3:**
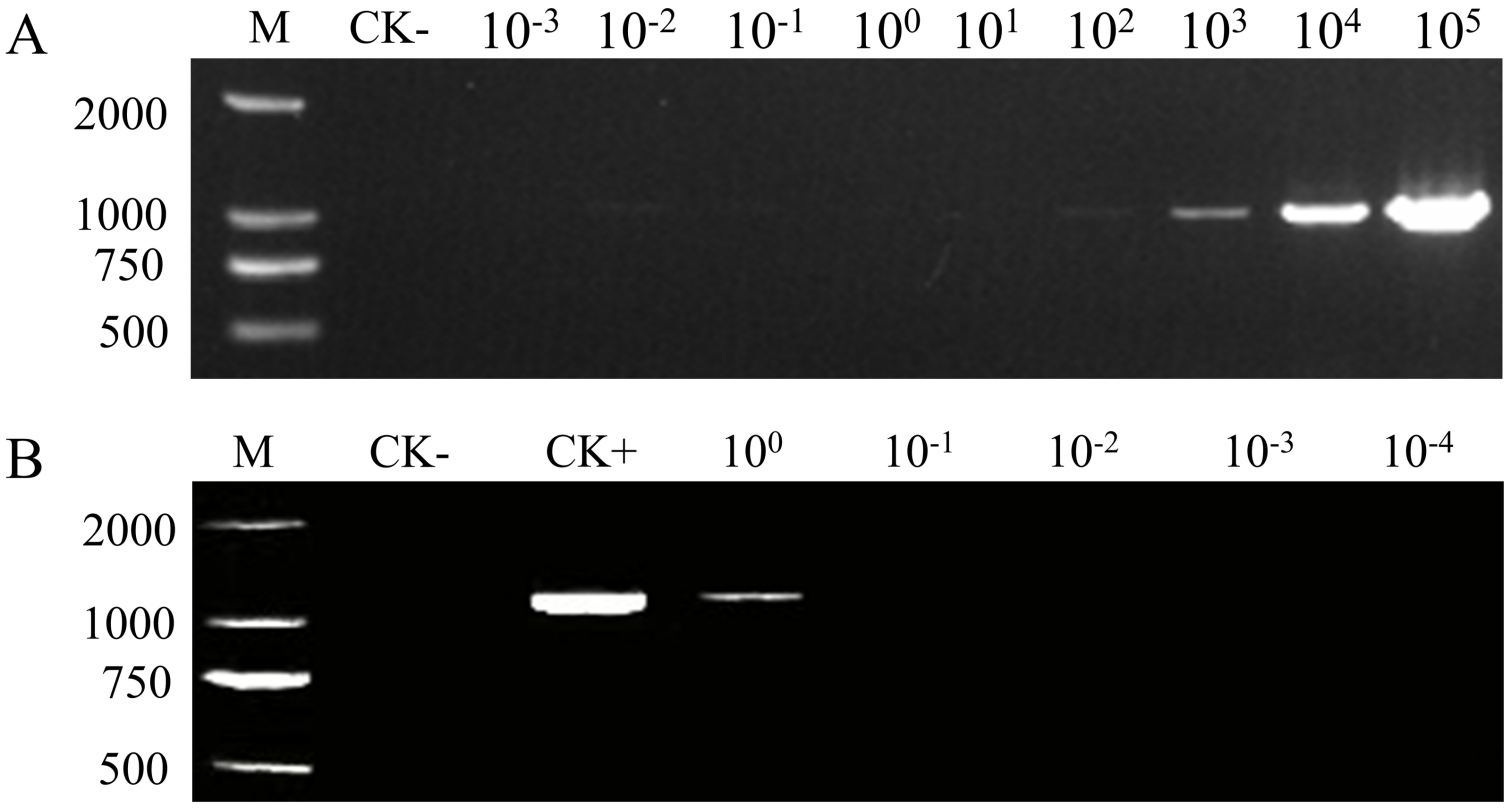
Sensitivity analysis of the PCR assay. M, DNA marker; CK-, PCR product amplified with ddH_2_O as a negative control template; CK+, PCR product amplified with pEASY-1138 bp as a positive control template. (A) PCR products amplified from the tenfold serial diluted plasmids, ranging from 2 × 10^−3^ copies/μL to 2 × 10^5^ copies/μL. (B) PCR products amplified from the tenfold serial dilution of DNA from a single larva, ranging from the equivalent of one larva down to 10^4^ of a larva. All templates were amplified with 32 cycles under the optimal PCR conditions in a 20 μL reaction system.

Under optimal PCR conditions in a 20 μL reaction system, the amplification products displayed a gradient reduction in gel band intensity that correlated with the decreasing template DNA copy numbers (Fig. 3A). The GroF1/GroR1 primer set detected plasmid DNA at levels as low as 2 × 10^2^ copies (1.11 fg) in this system, corresponding to a detection sensitivity of 10 copies/μL (5.6 × 10^−2^ fg/μL). The PCR sensitivity was also evaluated with diluted crude DNA extracted from a single larva of *G. rostochiensis* (Fig. 3B). The results reveal that the PCR assays can effectively detect DNA extracted from a single larva.

### Development of an RAA-based isothermal detection assay

**Figure 4:**
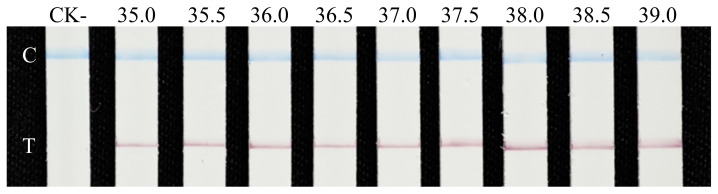
Optimization of the amplification temperature for the RAA-LFD assay. LFD detection results of RAA amplification at different temperatures; CK-, RAA product amplified with ddH_2_O as a negative control template; Strip 2–10: RAA products amplified at nine temperatures across 35–39 °C (0.5 °C intervals) for 30 min; C, control line; T, test line.

The primers RAA-GroF1/RAA-GroR1 and Gro-Probe were designed using Primer Premier 5.0 to target the *G. rostochiensis*-specific 1,138 bp fragment in plasmid pEASY-1138 bp (Table 1). Using *G. rostochiensis* genomic DNA as the template and ddH_2_O as a negative control, the primers and probe were validated. Primer pair RAA-GroF1/RAA-GroR1 successfully amplified the expected 199 bp target fragment. RAA amplification was tested across a temperature gradient (35–39 °C at 0.5  °C increments). LFD analysis revealed test lines at all temperatures, with quantitatively comparable signal intensities showing no significant differences (Fig. 4). Agarose gel electrophoresis confirmed the uniform amplification of the 199 bp target across all temperatures, with consistent band intensities. To minimize instrument dependency, 37.5 °C (close to human axillary temperature) was selected as the optimal amplification temperature for the RAA-LFD assay.

To establish the minimal amplification time required for effective detection with the RAA-LFD system, seven time points (5, 10, 15, 20, 25, 30, and 35 min) were tested at 5 min intervals. Subsequent analysis using LFD test strips revealed consistent positive results across all time points, as evidenced by the presence of a distinct red detection line (Fig. S2). Notably, no significant variation in detection line intensity was observed between different amplification durations. This finding was corroborated by agarose gel electrophoresis results, which demonstrated comparable band intensity for all amplification products. These results collectively indicate that when using high template concentrations, a remarkably short amplification period of just 5 min is sufficient to generate detectable products, enabling ultra diagnosis without compromising detection sensitivity.

**Figure 5:**
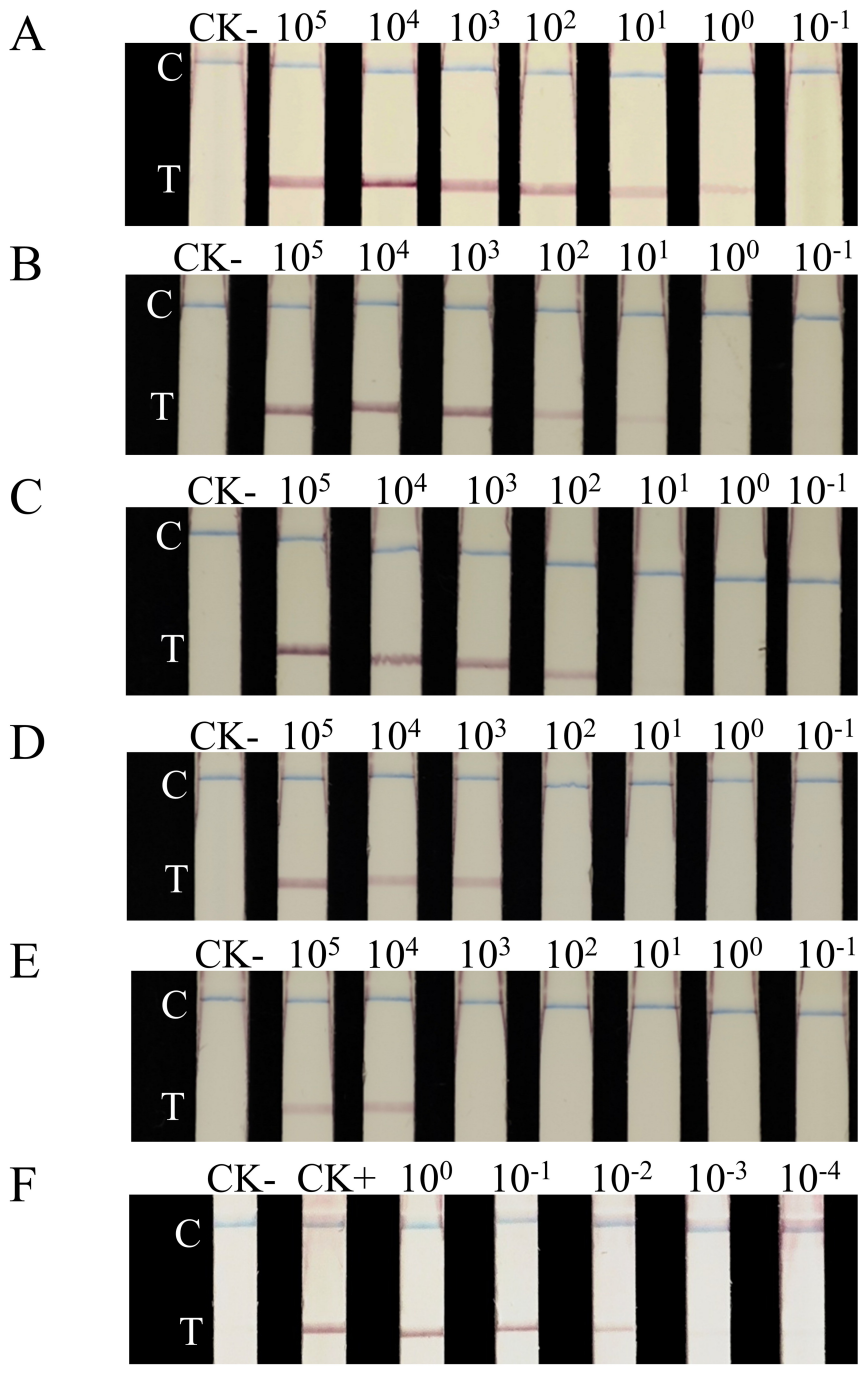
Sensitivity evaluation of the RAA-LFD assay. CK-, RAA product amplified with ddH_2_O as a negative control template; CK+, RAA product amplified with pEASY-1138bp as a positive control template; (A–E), LFD detection results of RAA amplification at different times; (A) 30 min; (B) 25 min; (C) 20 min; (D) 15 min; (E) 10 min; Strip 2–8: RAA products amplified with the tenfold serial diluted plasmids, ranging from 2 × 10^5^ copies/μL to 2 × 10^−1^ copies/μL. (F) sensitivity evaluation of the RAA-LFD assay using diluted single-larva DNA; Strip 3–7: RAA products amplified from the tenfold serial dilution of DNA from a single larva, ranging from the equivalent of one larva down to 10^−4^ of a larva. C, control line; T, test line.

The sensitivity of the RAA-LFD system improved progressively as the amplification time was extended (Fig. 5). The recombinant plasmid, initially set at a concentration of 2.3 × 10^10^ copies/µL, was first diluted to 2.3 × 10^5^ copies/µL, followed by 10-fold serial dilutions to generate a concentration gradient ranging from 10^5^ to 10^−1^ copies/µL. For the 25 µL RAA reaction system, the detection limit was determined as 5.75 × 10^4^ copies (321.4 fg) after 10 min of amplification (Fig. 5E). Following 15 min of amplification, the detection limit for the same 25 µL RAA reaction system decreased to 5.75 × 10^3^ copies (32.14 fg) (Fig. 5D). Similarly, the detection limits were further reduced to 5.75 × 10^0^ copies (0.03214 fg), 5.75 × 10^1^ copies (0.3214 fg), and 5.75 × 10^2^ copies (3.214 fg) following 30, 25, and 20 min amplification, respectively (Figs. 5A, 5B, 5C).

The RAA-LFD sensitivity was also evaluated with diluted crude DNA extracted from a single *G. rostochiensis* larva (Fig. 5F). The results reveal that under amplification at 37.5 °C for 30 min, the RAA-LFD assays can effectively detect the 10^−2^ dilution of DNA extracted from a single larva.

### Specificity evaluation of the PCR and RAA assays

**Figure 6:**
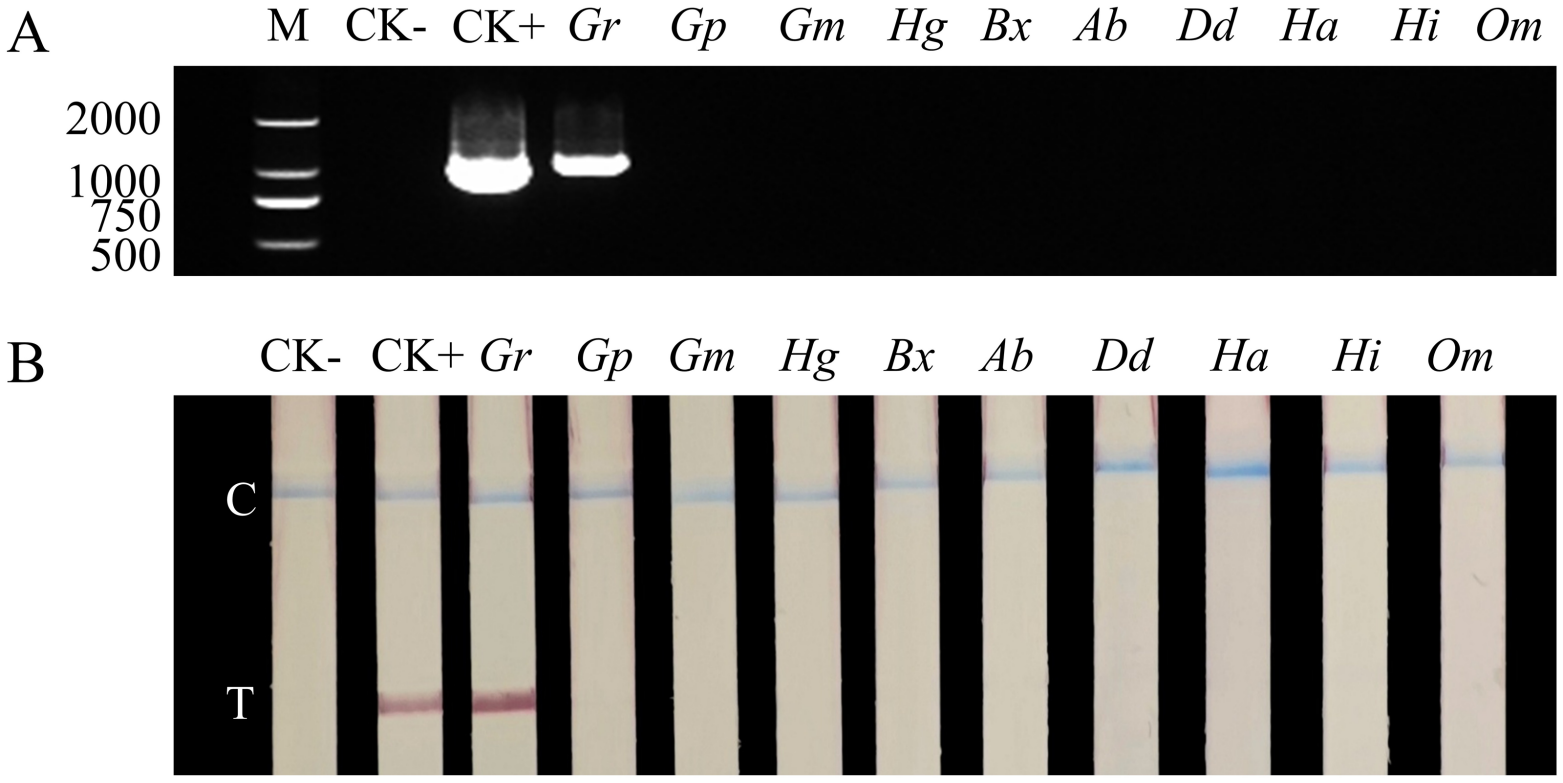
Specificity evaluation of PCR and RAA assays. (A) PCR products amplified with primer GroF and GroR1; M, DNA marker; (B) LFD detection results of RAA amplification; C, control line; T, test line. CK-, PCR or RAA product amplified with ddH_2_O as a negative control template; CK+, PCR or RAA product amplified with pEASY-1138bp as a positive control template; *Gr*, *G. rostochiensi*; *Gp*, *G. pallid*; *Gm*, *G. Mexicana*; *Hg*, *Heterodera glycines*; *Bx*, *Bursaphelenchus xylophilus*; *Ab*, *Aphelenchoides besseyi*; *Dd*, *Ditylenchus destructor*; *Ha*, *Heterodera avenae*; *Hi*, *Heterorhabditis indica*; *Om*, *Oscheius myriophilus*.

The specificity of the PCR and RAA-LFD assays was assessed using genomic DNA of *G. rostochiensis* and nine other nematode species, including *G. pallida*, *G. mexicana*, *Heterodera glycines*, *Bursaphelenchus xylophilus*, *Aphelenchoides besseyi*, *Ditylenchus destructor*, *Heterodera avenae*, *Heterorhabditis indica*, and *Oscheius myriophilus*. The PCR and RAA-LFD results both indicate high specificity for *G. rostochiensis*. Only the *G. rostochiensis* samples generated a distinct electrophoretic band (Fig. 6A) or LFD test line (Fig. 6B), while electrophoretic bands and visible test lines were not present in other cyst-nematode species or in the negative control. These results indicate that both the PCR and RAA-LFD assays can specifically distinguish *G. rostochiensis* from other nematode species.

### Detection of *G. rostochiensis* in field soil samples

**Figure 7:**
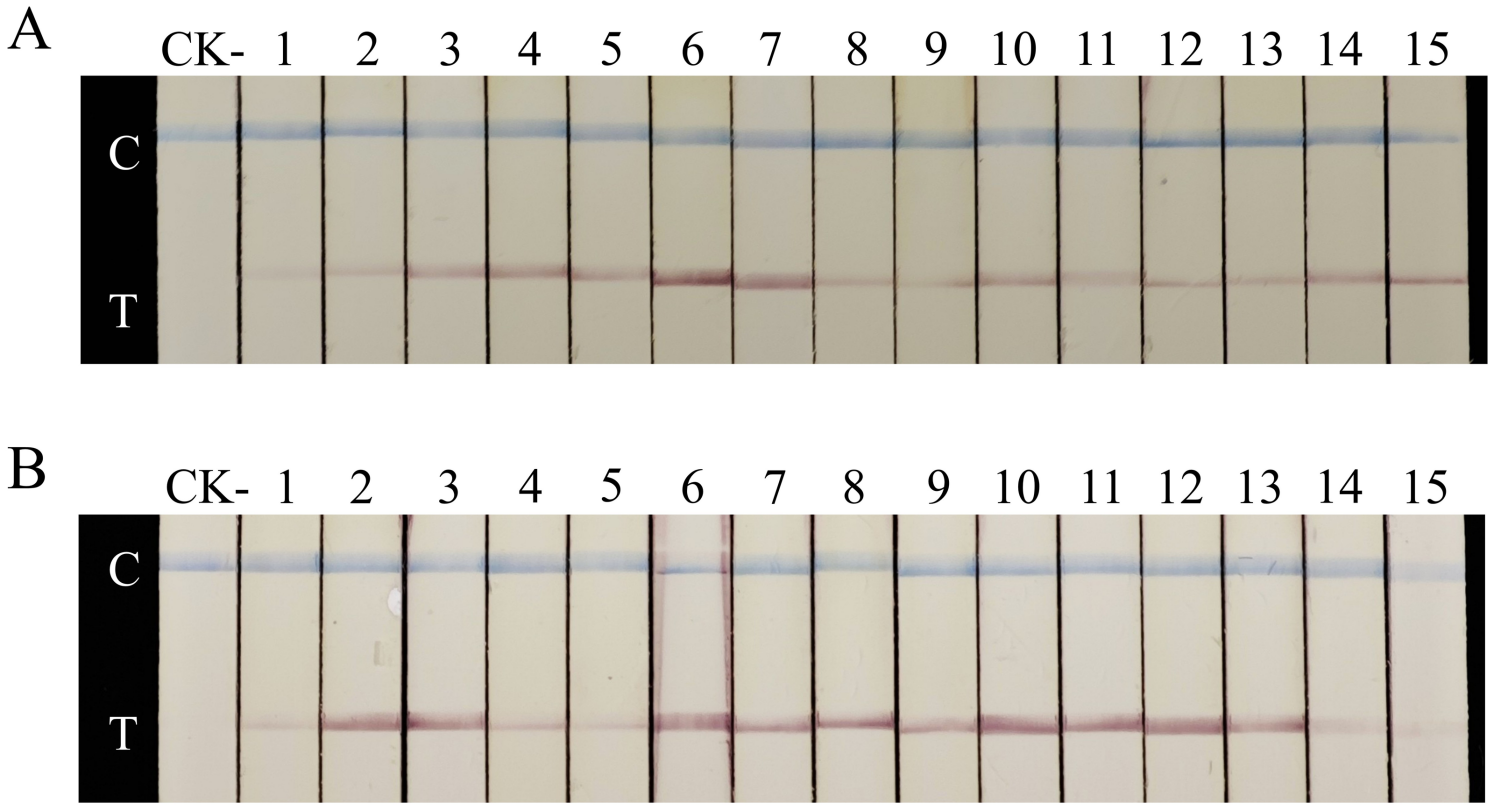
Detection of *G. rostochiensis.*-infested soil samples by the RAA-LFD assay. LFD detection results of RAA amplification at different times; (A) 30 min; (B) 25 min; CK-, RAA products amplified using *G. rostochiensis*-free soil DNA as a negative control; Strip 2–16, infested soil samples; C, control line; T, test line.

Genomic DNA extracted from 15 soil samples collected within *G. rostochiensis* quarantine zones was confirmed to contain *G. rostochiensis* DNA using the detection method specified in the Industry Standard of the People’s Republic of China for Entry-Exit Inspection and Quarantine (SN/T 1723.1-2022, Fig. S3). All 15 DNA samples successfully amplified the target band with the GroF1/GroR1 primer set in the PCR detection system. Subsequent testing using the RAA assay with RAA-GroF1/RAA-GroR1 primers and Gro-Probe yielded a 100% positive detection rate across all infested soil samples at the 25- and 30-min amplification (Fig. 7). However, faint test lines persisted even after 30 min of amplification in several samples, indicating low *G. rostochiensis* concentrations in the soil. Consequently, shorter amplification times were not evaluated.

## Discussion

Traditional morphological identification of *G. rostochiensis* suffers from suboptimal accuracy, time inefficiency, and operational complexity, rendering it impractical for rapid field detection (Subbotin et al., 2010; Viney & Diaz, 2012; Bogale, Baniya & Di Gennaro, 2020; Wainer & Dinh, 2021; Perry & Wright, 1998). In contrast, PCR-based detection technologies (*e.g.*, qPCR, multiplex PCR, and PCR-RFLP) offer rapid, highly sensitive, and species-specific detection (Thiery & Mugniery, 1996; Bulman & Marshall, 1997; Mwangi et al., 2015; White et al., 1990; Kralik & Ricchi, 2017; EPPO Standard on Diagnostics, 2022).The establishment of PCR detection methods critically depends on the selection of appropriate molecular targets, the design of highly specific primers, the optimization of amplification conditions, and the minimization of primer-dimer formation (Czerny, 1996; LaMontagne et al., 2002). Despite numerous reported molecular detection systems, alignment against the NCBI database reveals notable specificity deficiencies in published primers/targets, particularly regarding discrimination from congeneric species. For example, the forward primer (5′-CCCTGACCGAATTCTGTT-3′) in Peng’s patent (Peng, Ou & Peng, 2014) fully matches with several genome regions of *Catalinimonas niigatensis*, *Paracidovorax wautersii strain* and *Salmonella enterica*, and is highly similar (>16 bp) to genome regions of *Polyprion americanus*, *Seriola dumerili*, *Lotoria lotoria*, *Erythrolamprus reginae*, *Hylaeus volcanicus, etc*. The reverse primer (5′-AAATTGTCGGACGCGAAT-3′) fully matches with several genome regions of *Parageobacillus thermoglucosidasius*, *Candidatus Poribacteria*, *Breznakiella homolactica*, and *Mycolicibacterium neoaurum*, as well as other microbes (Table 2). We noted that the forward and reverse primers described in Ge’s patent (Ge et al., 2009b) may not exhibit strict specificity for *G. rostochiensis*. The forward primer (5′-ACTCCATGTTGTACGTGCCG-3′) also fully matches with several uncharacterized *Globodera* species, while the reverse primer (5′-CACGGCCACGGACGTAG-3′) is completely identical to sequences from *G. pallida*, several *Heterodera* species, as well as species belonging to the genera *Punctodera* and *Cactodera* (Table 2). Similarly, the forward primer (5′-CTGTGTATGGGCTGGCACATTGACCAACA-3′) and the reverse primer (5′-biotin-TACGGCACGTACAACATGGAGTAGCAGCTAC-3′) employed in Wang et al. (2022b) also align closely with several uncharacterized *Globodera* species (Table 2). These observeations highlight a critical limitation: if these primer sets are used in assays involving complex field samples, there is a considerable likelihood of non-specific amplification, leading to potentially false-positive results.

**Table 2:** *In silico* alignment of published *G. rostochiensis* primer sequences against non-target organisms (NCBI nt).

Primers	Sequence (5′→3′)	Top non-target matches	Identities
GrF3 (Peng, Ou & Peng,2014)	CCCTGACCGAATTCTGTT	*Paracidovorax wautersi* *Salmonella enteric* *Catalinimonas niigatensi*	18/18
GrB3 (Peng, Ou & Peng, 2014)	AAATTGTCGGACGCGAAT	*Parageobacillus thermoglucosidasius* *Candidatus Poribacteria* *Breznakiella homolactica* *Mycolicibacterium neoaurum* *Tytthaspis sedecimpunctata*	18/18
SEQ ID No.1 (Ge et al., 2009b)	ACTCCATGTTGTACGTGCCG	*Globodera* sp.	20/20
SEQ ID No.2 (Ge et al., 2009b)	CACGGCCACGGACGTAG	*Heterodera latipons* *Heterodera glycines* *Heterodera salixophila* *Punctodera mulveyi* *Cactodera torreyanae* *Heterodera medicaginis* *Heterodera schachtii* *Heterodera filipjevi* *Globodera pallida* *Heterodera pratensis*	17/17
GrF4 (Wang et al., 2022a)	CTGTGTATGGGCTGGCACATTGACCAACA	*Globodera* sp.	29/29
GrR4 (Wang et al., 2022a)	Biotin-TACGGCACGTACAACATGGAGTAGCAGCTAC	*Globodera* sp.	31/31

In this study, target DNA was identified through comprehensive comparative genomics analysis, revealing the 3,572 bp *G. rostochiensis*-specific fragments with no significant matches to any database sequences. The target DNA is considered strictly species-specific, and the designed PCR and RAA primers are therefore expected to exhibit high specificity. However, the currently available genome assemblies of *G. rostochiensis* in database remain somewhat fragmented and incomplete characterized, for example, by the presence of numerous scaffolds and gaps. This has the potential to compromise the reliability of the target sequence for species-specific detection. Our alignment analyses indicate that the PCR forward primer shares more than 18 bp of highest similarity with non-nematode animal species, including *Chloris chloris*, *Spinus* sp., *Fringilla* coelebs, *Pungitius* sp., and *Notodromas monacha*. Given that these are birds, fish, or crustaceans—rather than nematodes—we consider it unlikely that these in silico matches would compromise assay specificity in a nematode diagnostic context. In addition, the PCR reverse primer aligns over more than 16 bp with *Sulfitobacter mediterraneus*, *Hwanghaeella* sp., *Demequina aurantiaca*, and *Methylotuvimicrobium* sp. The RAA forward primer shows more than 19 bp similarity with *Camellia sinensis*, *Poecilia reticulata*, *Notodromas monacha*, *Luscinia svecica*, and *Apteryx australis*, while the RAA reverse primer shares over 19 bp with *Brassica oleracea*, *B. napus*, *B. rapa*, *Ascaphus truei*, *Raphanus sativus*, and *Cairina moschata*. Since none of these species belong to the nematode phylum, we conclude that the risk of cross-amplification in our nematode detection assay is negligible. This is the critical advantage absent in previously reported systems.

High sensitivity is also a critical metric in molecular diagnostics, reflecting the capacity of an established PCR assay to reliably detect low DNA concentrations in samples. In this study, the primer pair GroF1/GroR1 demonstrated a sensitivity of 10 copies/μL (5.6 ×10^−2^ fg/μL) after 32 PCR cycles in a 20 μL reaction system. The sensitivity of the RPA-LFD (Wang et al., 2022b) and RPA-LFA (Wang et al., 2022a) assays reached 1 × 10^−3^ single cysts. However, this estimation assumes complete DNA extraction from single cysts. In practical experiments, inevitable DNA loss during extraction may result in lower actual sensitivity than predicted. Ahuja et al. (2021) constructed a PCN-LAMP assay system for *G. rostochiensis* with a sensitivity of 1 fg/μL. Moreover Van de Vossenberg et al. (2014) achieved *G. rostochiensis* detection using conventional PCR, with a sensitivity of a single DNA molecule extracted from a single larva, approximately from 1 × 10^4^ to 1 × 10^6^ copies/μL. Ge et al. (2009a) applied TaqMan probes for the real-time fluorescent PCR detection of *G. rostochiensis*, with the MJTS/MJTX primers exhibiting a sensitivity of 10 fg/μL. Toyota et al. (2008) developed a real-time PCR assay sensitive to DNA extracted from a single second-stage juvenile (J2), approximately corresponding to the range from 1 × 10^4^ to 1 × 10^6^ copies/μL. The sensitivity of the GroF1/GroR1 primers used in this study outperforms the previously reported maximum sensitivity of 10 fg/μL in PCR-based molecular detection systems (Ge et al., 2009a), and is comparable to the PCN-LAMP technique (Ahuja et al., 2021).

However, PCR-based detection assays for *G. rostochiensis* remain constrained by their reliance on precise instrumentation and prolonged processing times. To address these limitations, RAA primers and a probe were designed using GroF1/GroR1 amplification products as molecular targets. A highly sensitive isothermal nucleic acid detection assay was subsequently developed through optimization and the integration with LFD visualization technology (Bian et al., 2022; Song, Cao & Yan, 2024; Forget & Kowalczykowski, 2012; Daher et al., 2016; Wang, Wang & Hou, 2019). The approach eliminates the necessity for colorimetric interpretation, providing more intuitive results *via* test strips. Jiao et al. (2024) designed RPA-specific primers and an EXO fluorescent probe targeting the ITS sequence of *G. rostochiensis*, reporting an RPA-EXO detection method with a sensitivity of 10^−3^ single cysts (10–40 copies/μL) after 30 min amplification at 38  °C. However, their probe exhibits notable sequence similarity (94.12%) to a region on the *G. tabacum* genome, risking cross-species detection. Furthermore, the method requires fluorescence thermocyclers for exonuclease-dependent signal generation and specialized instrumentation for the interpretation of the results.

Wang et al. (2022a) developed a RPA-LFA assay, targeting the internal transcribed spacer (ITS rDNA) of *G. rostochiensis*. The system effectively detected as little as 10^−1^ of a single juvenile from crude extracts within 30 min, and specifically distinguished *G. rostochiensis* from five other plant-parasitic nematode species. Subbotin (2025) designed an RPA-LFD assay for *G. rostochiensis* based on microsatellite markers. The method achieved a sensitivity equivalent to 0.03 of a single juvenile and could specifically detect *G. rostochiensis* among three other nematode species. Although the techniques reported in both studies demonstrate very high sensitivity and species specificity, they do not clearly indicate the number of detectable DNA copies. Thus, it remains unclear whether such sensitivity can be maintained under field conditions—with sample damage, DNA degradation, inhibitors, and other real-world challenges present. Moreover, the number of DNA copies varies among juvenile individuals. Additionally, the efficiency of DNA extraction is influenced by the specific experimental protocols. The RAA-LFD detection assay developed in this study demonstrates exceptional sensitivity, detecting as few as 57.5 copies (2.3 copies/μL) following 25 min amplification at 37.5 °C. This sensitivity increased to 5.75 copies (0.23 copies/μL) with 30 min amplification, with both conditions achieving 100% detection rates in field-infected potato soil samples. Under sufficient amplification time (37.5  °C, 30 min), the RAA-LFD assay can reliably detect a 10^−2^ dilution of DNA extracted from a single larva. The RAA-LFD assay developed in this study features dual calibration through probe-antibody verification, significantly reducing cross-reactivity risks. The RAA-LFD assay is able to specifically distinguish *G. rostochiensis* from nine other nematode species, demonstrating excellent specificity. Test strips avoid enzymatic inhibition steps, enhancing interference resistance, while enabling naked-eye interpretation. This system is optimized for rapid field screening, with particular applicability in grassroots agricultural settings and quarantine stations. The complete workflow—including DNA extraction, RAA amplification (25–30 min at 37.5 °C), and LFD detection—requires approximately one hour. Compared to existing detection platforms, this approach demonstrates superior field applicability through faster processing times, enhanced sensitivity (detection limit: 0.23–2.3 copies/μL), reduced operational costs, and minimal instrumentation requirements. While the RAA-LFD assay developed in this study exhibited high sensitivity and operational convenience, it still has certain limitations in terms of specificity compared with previously reported RPA-LFD methods (Wang et al., 2022a and Subbotin, 2025). Specificity evaluation in this work was confined to a limited panel of non-target nematode species. In the future, as more new species (particularly those that are closely related) are discovered and their genomes are reported, perhaps our detection assays will no longer possess strong species specificity. Further validation across a wider spectrum of phylogenetically related taxa remains necessary to confirm its robustness. As regulated quarantine pests, obtaining overseas samples and securing import approval are exceptionally challenging. Consequently, to maximize species specificity within the limits of current knowledge, the primers and probe were aligned against published genome assemblies and nucleotide sequences of relevant species. Future efforts should therefore aim to expand the range of non-target organisms tested to further improve both assay specificity and practical reliability.

## Conclusions

A 3,572 bp *G. rostochiensis*-specific DNA fragment, and PCR- and RAA-based diagnostic assays were developed accordingly. The RAA-LFD and PCR assays can effectively distinguish *G. rostochiensis* from nine other pathogenic nematodes. The detection limit of the PCR assay was 200 copies when amplified over 32 cycles within the optimized conditions. The optimized RAA assay exhibited detection limits of 5.75 × 10^2^, 5.75 × 10^1^, and 5.75 × 10^0^ copies after 20, 25, and 30 min amplification, respectively, at 37.5 °C. The PCR assay was able to accurately detect a single larva, whereas the RAA-LFD detected a 10^−2^-fold dilution of DNA from a single larva. The positive detection rate of infested soil samples reached 100% following a 25 min isothermal amplification at 37.5  °C. In summary, PCR- and RAA-based rapid and highly sensitive detection assay were developed for *G. rostochiensis*.

## Supplemental Information

10.7717/peerj.21155/supp-1 Supplemental Information 1 Optimization of the annealing temperature for the PCR amplification reaction M: DNA marker; Lane 2–9: PCR products amplified at different temperatures, spanning from 57.0 °C to 62.0 °C; CK-: PCR product amplified with ddH_2_O as a negative control template.

10.7717/peerj.21155/supp-2 Supplemental Information 2 Optimization of the amplification time for the RAA amplification reaction LFD detection results of RAA amplification at different times; CK-: RAA product amplified with ddH_2_O as a negative control template; Strip 2–8: 5, 10, 15, 20, 25, 30, and 35 min; C: control line; T: test line.

10.7717/peerj.21155/supp-3 Supplemental Information 3 *G. rostochiensis* detection in soil samples using the method specified in the i ndustry s tandard of the People’s Republic of China M: DNA marker; CK-: PCR product amplified with ddH_2_O as a negative control template ; Lane 3–1 7: infested soil samples.

10.7717/peerj.21155/supp-4 Supplemental Information 4 Uncropped Figures

## Additional Information and Declarations

### Data Availability

The following information was supplied regarding data availability: The raw data are available in the Supplemental Files.
