# Supplementary figures and images for: Development of nucleic acid testing technology for quarantine pest Globodera rostochiensis based on a species-specific DNA fragment
Source: PeerJ. 2026 May 15;14:e21155. doi: 10.7717/peerj.21155 (PMC13182724; doi:10.7717/peerj.21155)

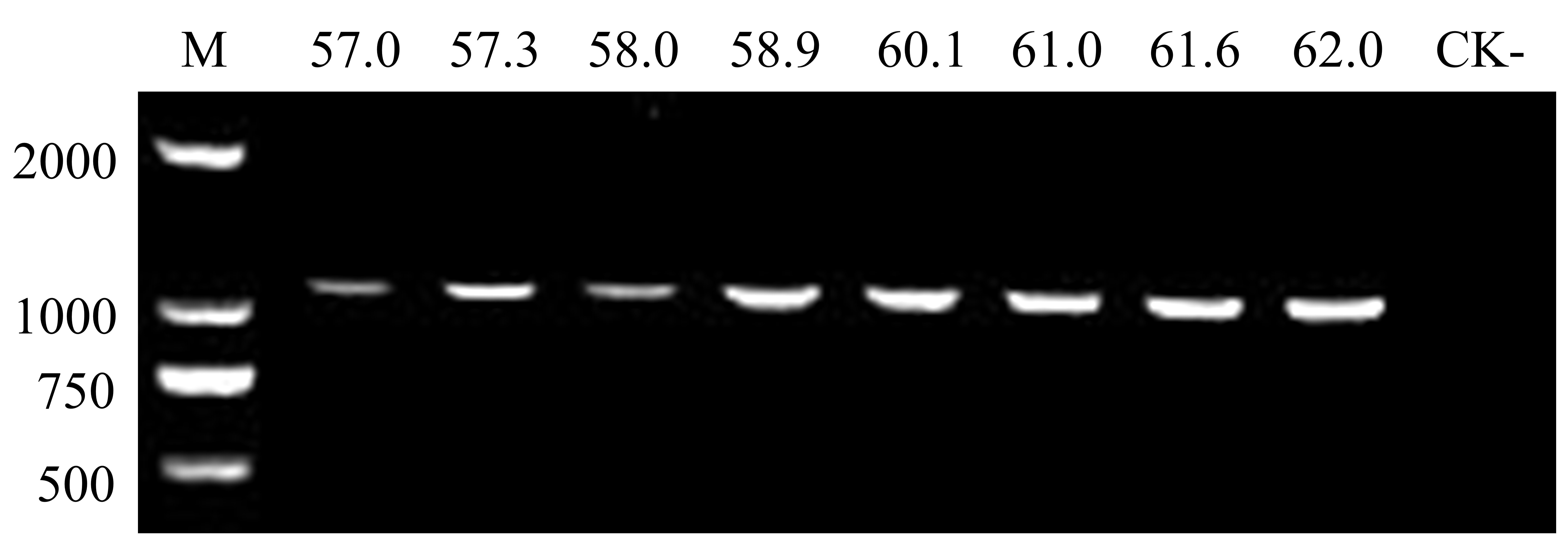

Supplement: Supplemental Information 1 — M: DNA marker; Lane 2–9: PCR products amplified at different temperatures, spanning from 57.0 °C to 62.0 °C; CK-: PCR product amplified with ddH2O as a negative control template. [file peerj-14-21155-s001.jpg]

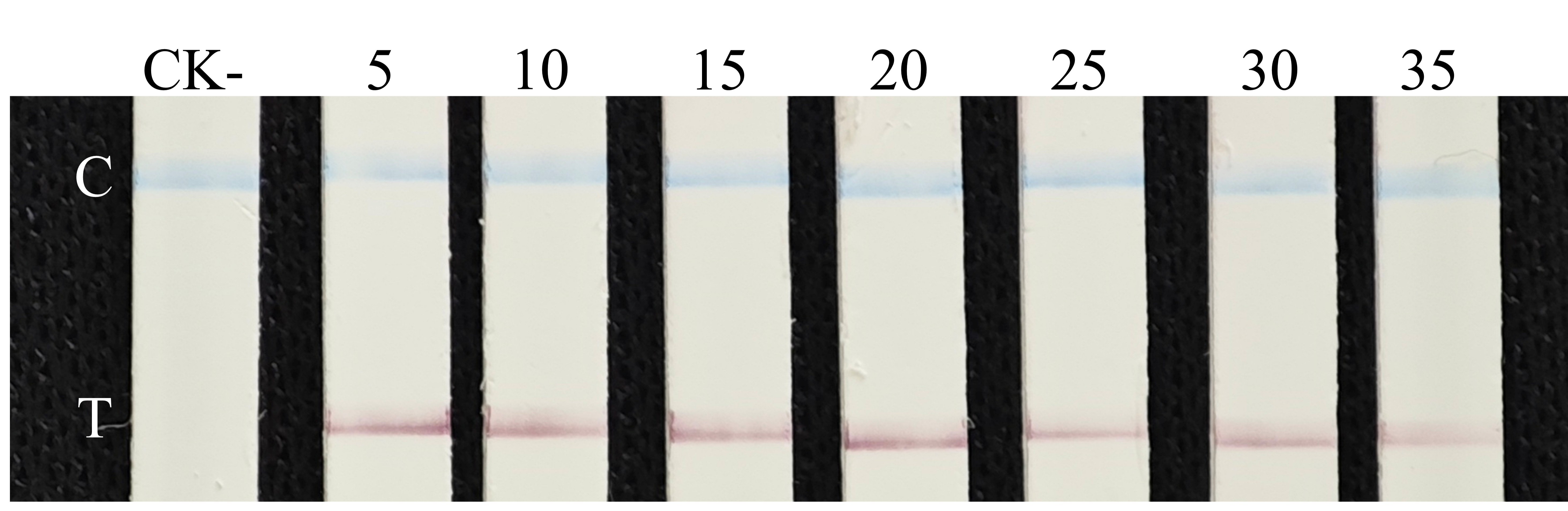

Supplement: Supplemental Information 2 — LFD detection results of RAA amplification at different times; CK-: RAA product amplified with ddH2O as a negative control template; Strip 2–8: 5, 10, 15, 20, 25, 30, and 35 min; C: control line; T: test line. [file peerj-14-21155-s002.jpg]

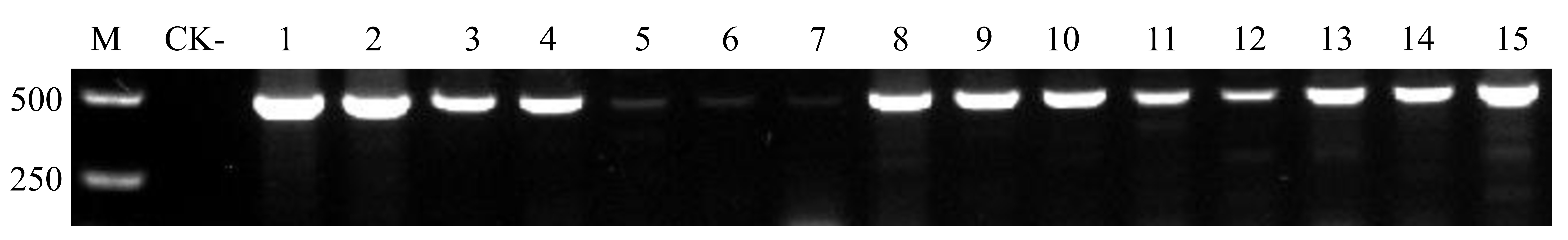

Supplement: Supplemental Information 3 — M: DNA marker; CK-: PCR product amplified with ddH2O as a negative control template ; Lane 3–1 7: infested soil samples. [file peerj-14-21155-s003.jpg]

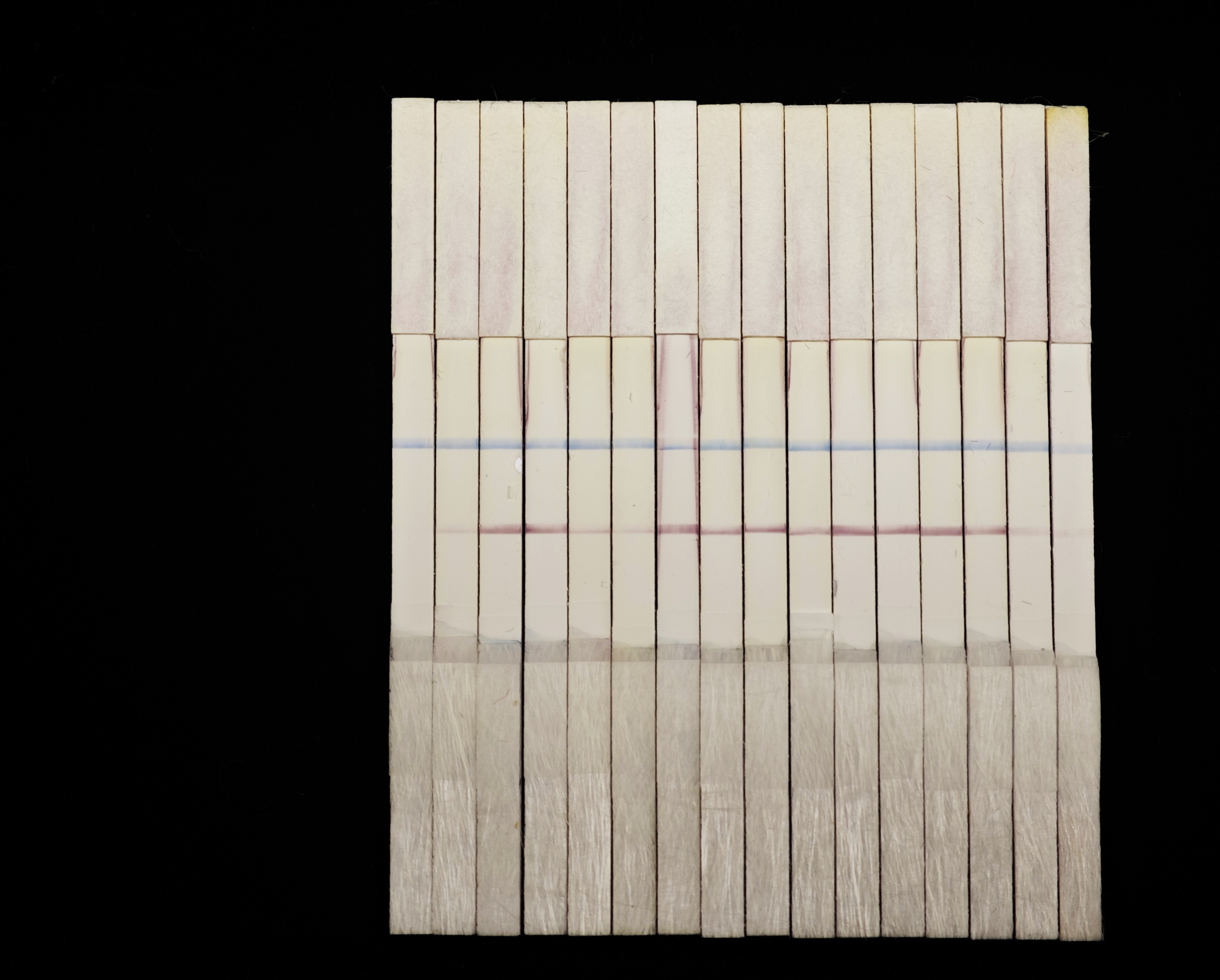

Supplement: Supplemental Information 4 [file peerj-14-21155-s004.zip › Uncropped figures/Detection of G. rostochiensis-infested soil samples by the RAA-LFD assay-25min.jpg]

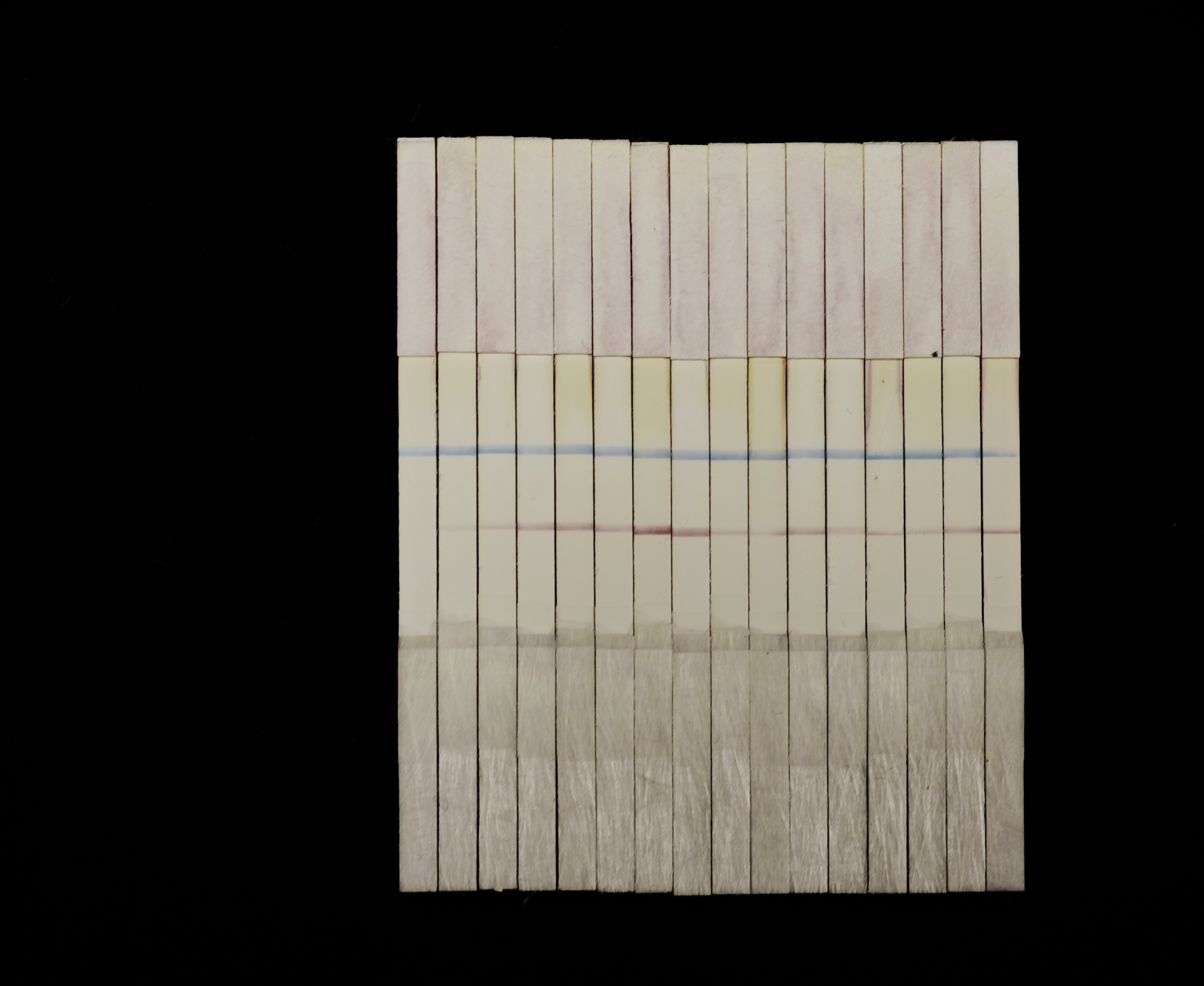

Supplement: Supplemental Information 4 [file peerj-14-21155-s004.zip › Uncropped figures/Detection of G. rostochiensis-infested soil samples by the RAA-LFD assay-30min.jpg]

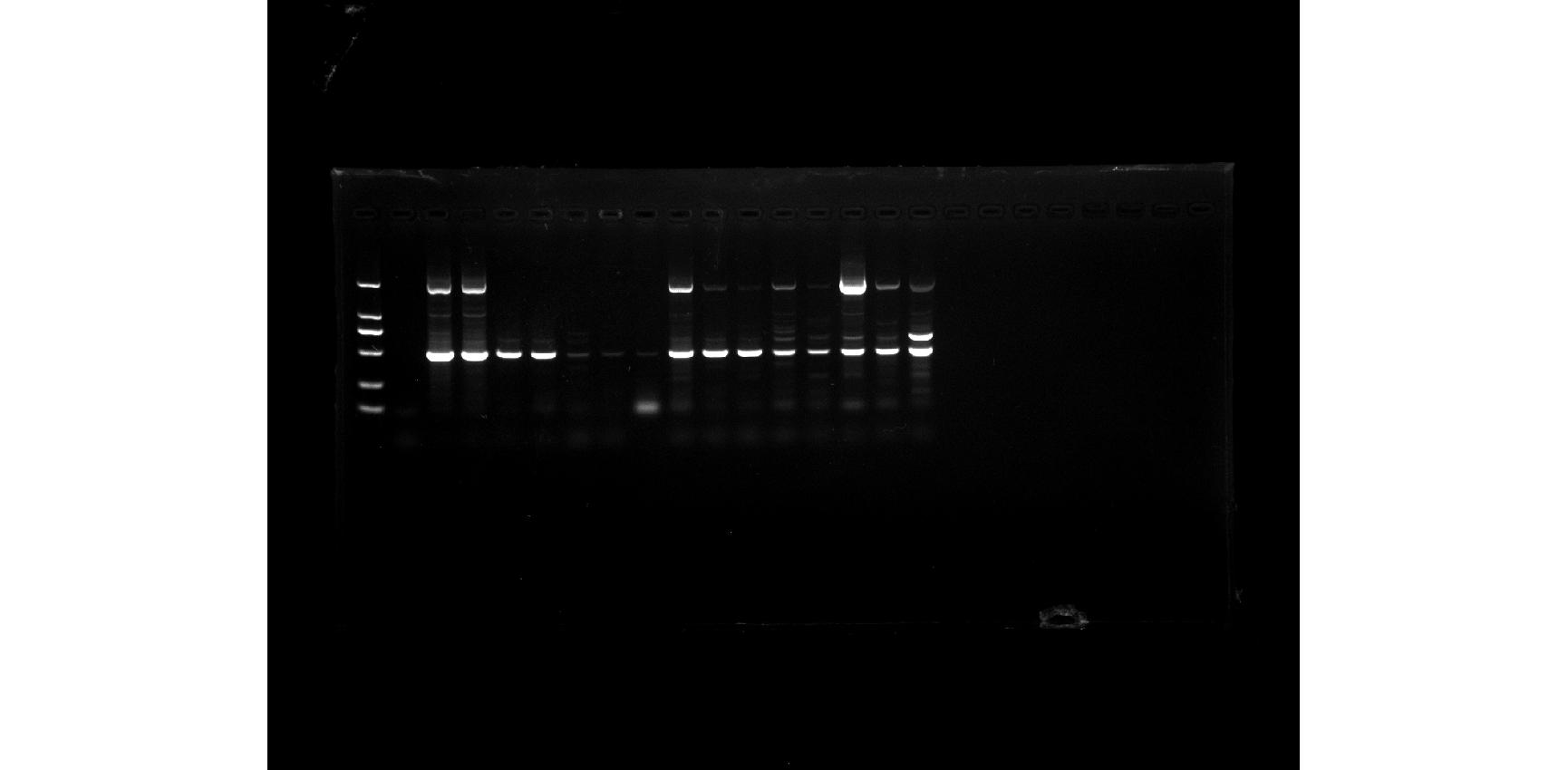

Supplement: Supplemental Information 4 [file peerj-14-21155-s004.zip › Uncropped figures/G. rostochiensis detection in soil samples using the method specified in the industry standard.jpg]

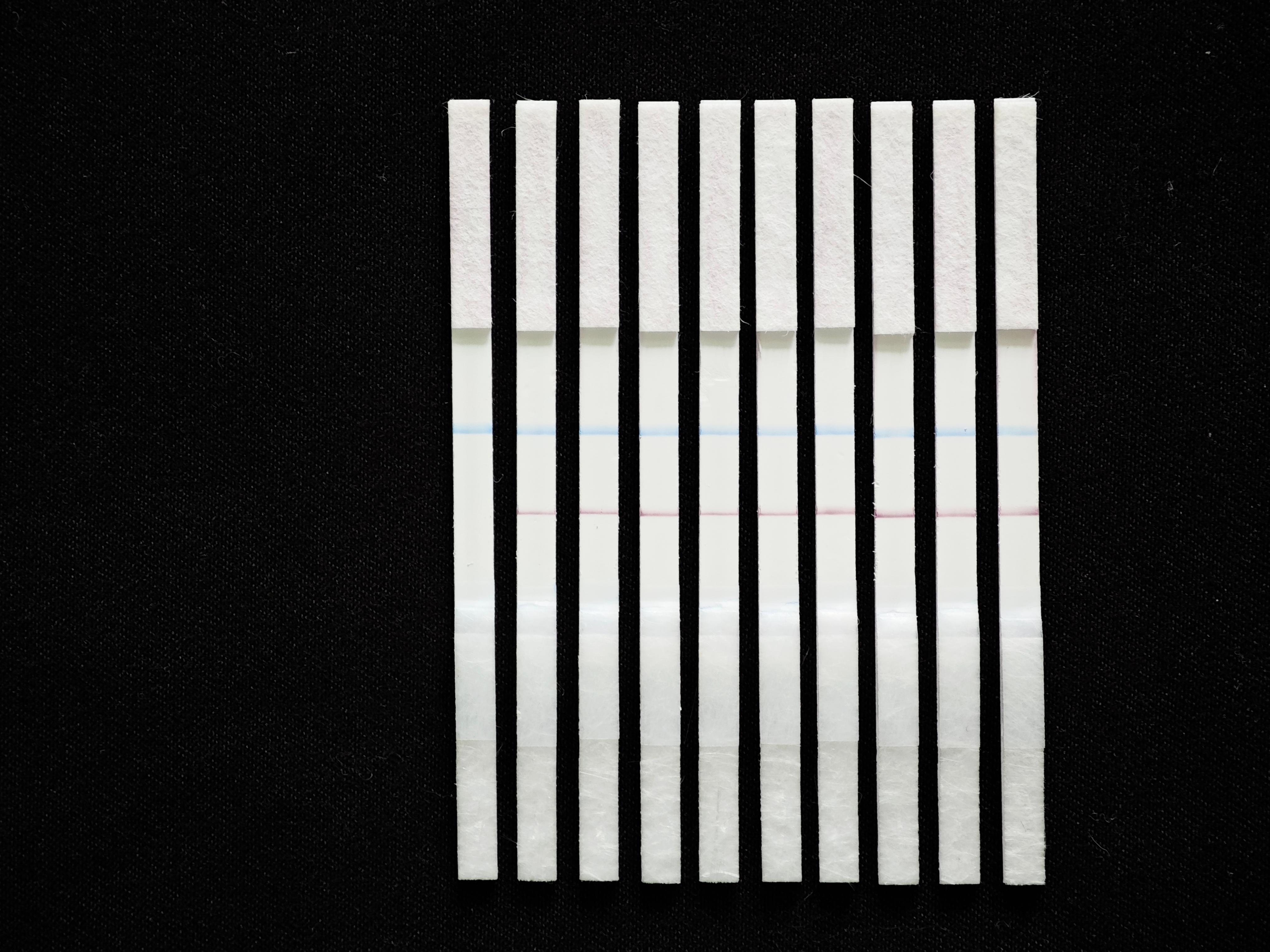

Supplement: Supplemental Information 4 [file peerj-14-21155-s004.zip › Uncropped figures/Optimization of amplification temperature for the RAA-LFD.jpg]

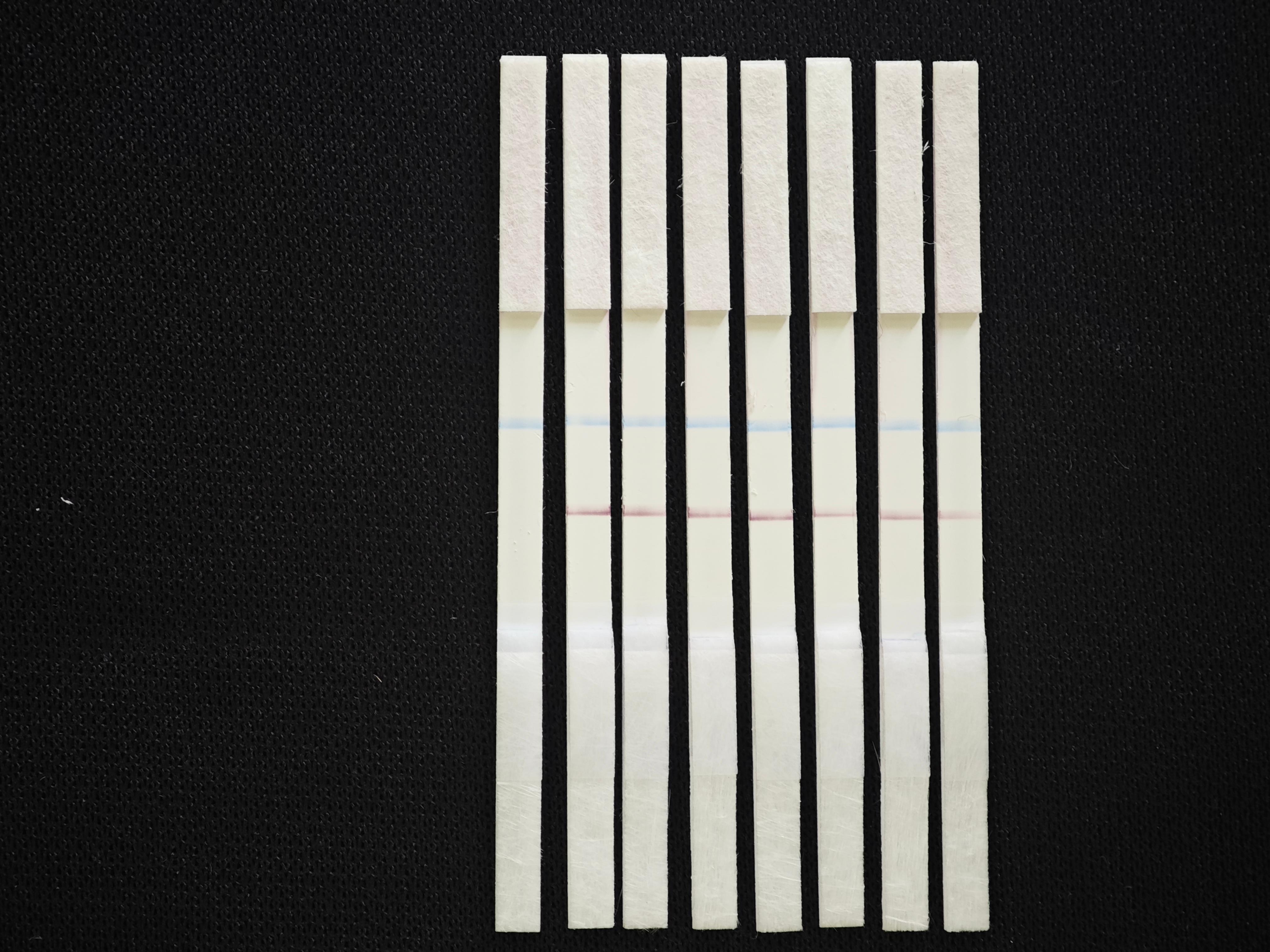

Supplement: Supplemental Information 4 [file peerj-14-21155-s004.zip › Uncropped figures/Optimization of amplification time for the RAA amplification reaction.jpg]

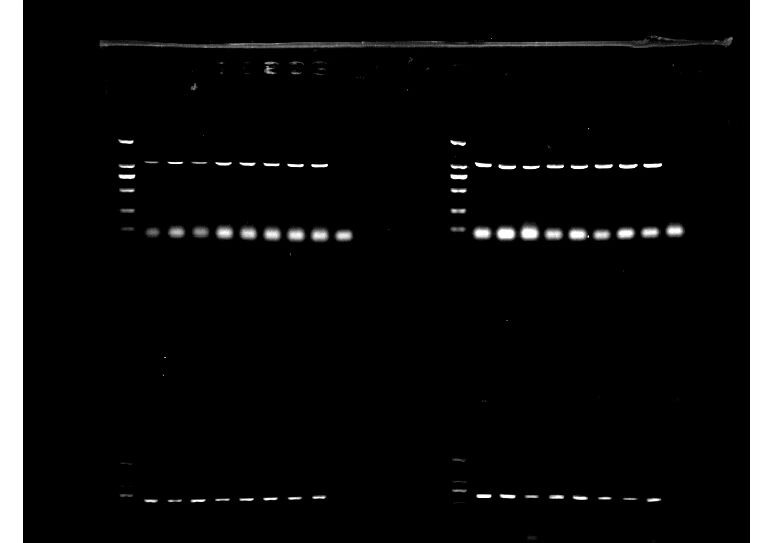

Supplement: Supplemental Information 4 [file peerj-14-21155-s004.zip › Uncropped figures/Optimization of annealing temperature.jpg]

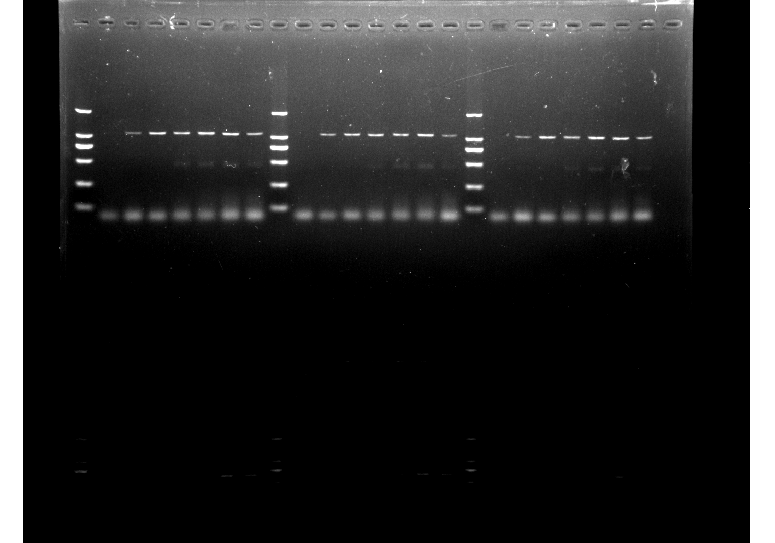

Supplement: Supplemental Information 4 [file peerj-14-21155-s004.zip › Uncropped figures/Optimization of cycle number.png]

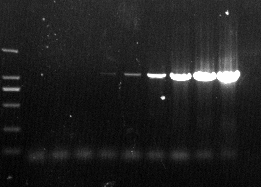

Supplement: Supplemental Information 4 [file peerj-14-21155-s004.zip › Uncropped figures/Optimization of template concentration 3.png]

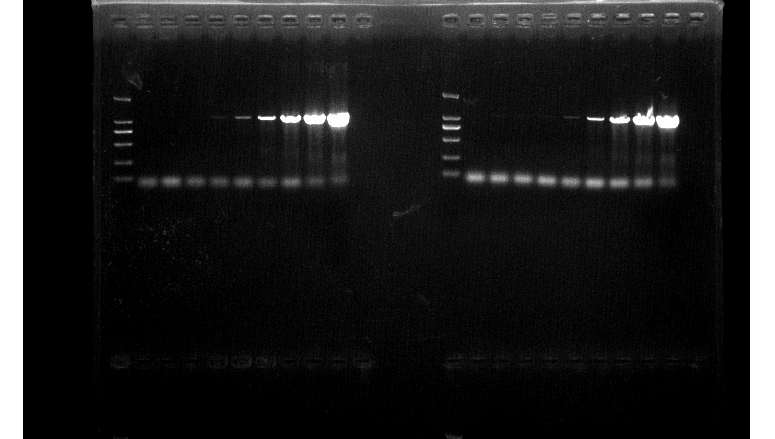

Supplement: Supplemental Information 4 [file peerj-14-21155-s004.zip › Uncropped figures/Optimization of template concentration.png]

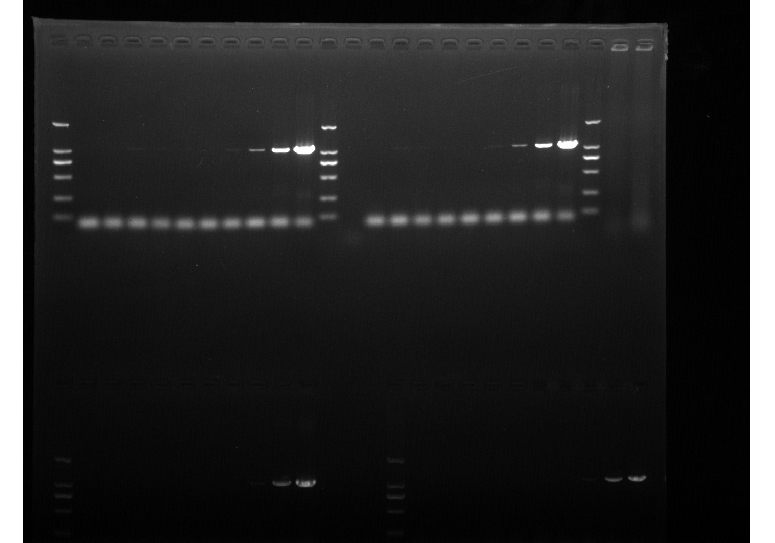

Supplement: Supplemental Information 4 [file peerj-14-21155-s004.zip › Uncropped figures/Sensitivity analysis of the PCR assay.png]

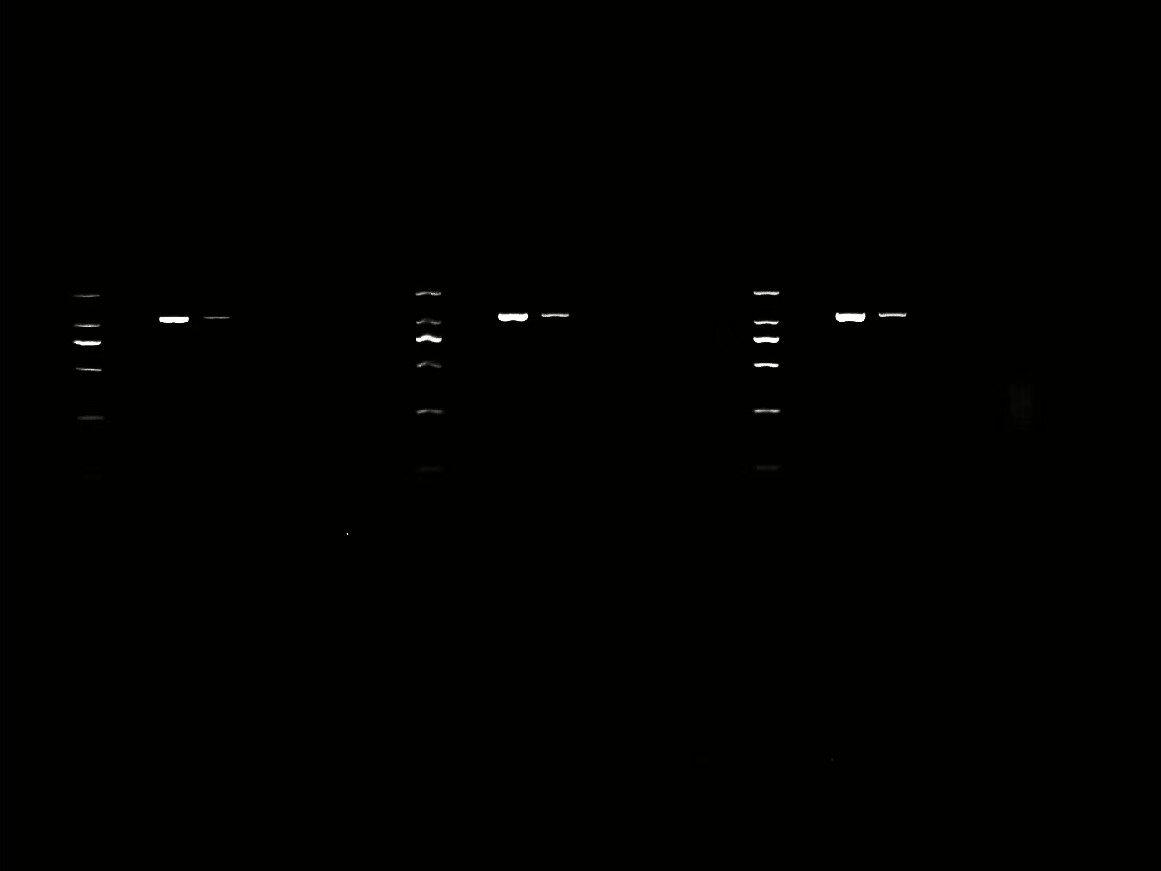

Supplement: Supplemental Information 4 [file peerj-14-21155-s004.zip › Uncropped figures/Sensitivity evaluation of the PCR assay using diluted single-larva DNA.jpg]

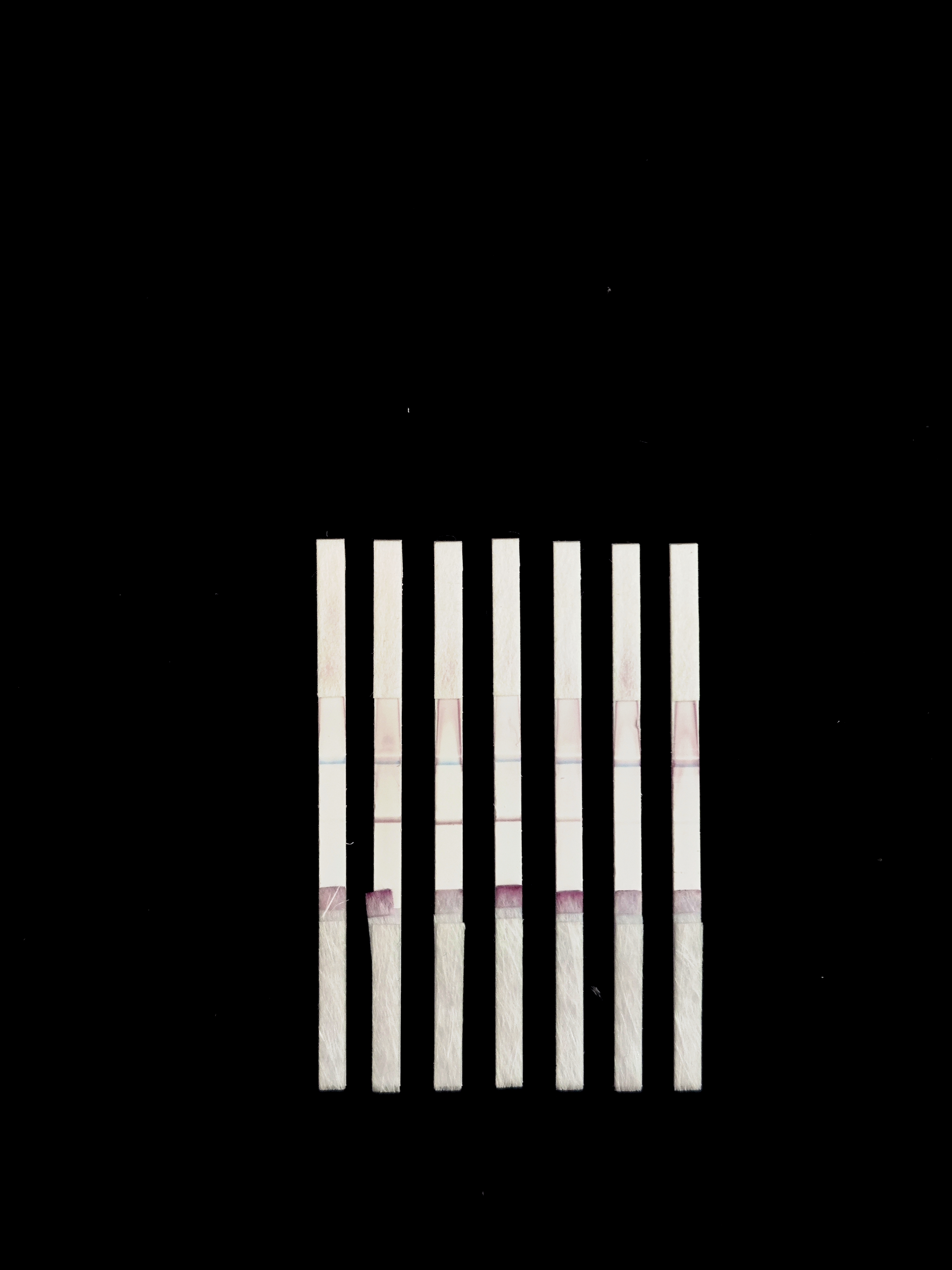

Supplement: Supplemental Information 4 [file peerj-14-21155-s004.zip › Uncropped figures/Sensitivity evaluation of the RAA-LFD assay using diluted single-larva DNA.jpg]

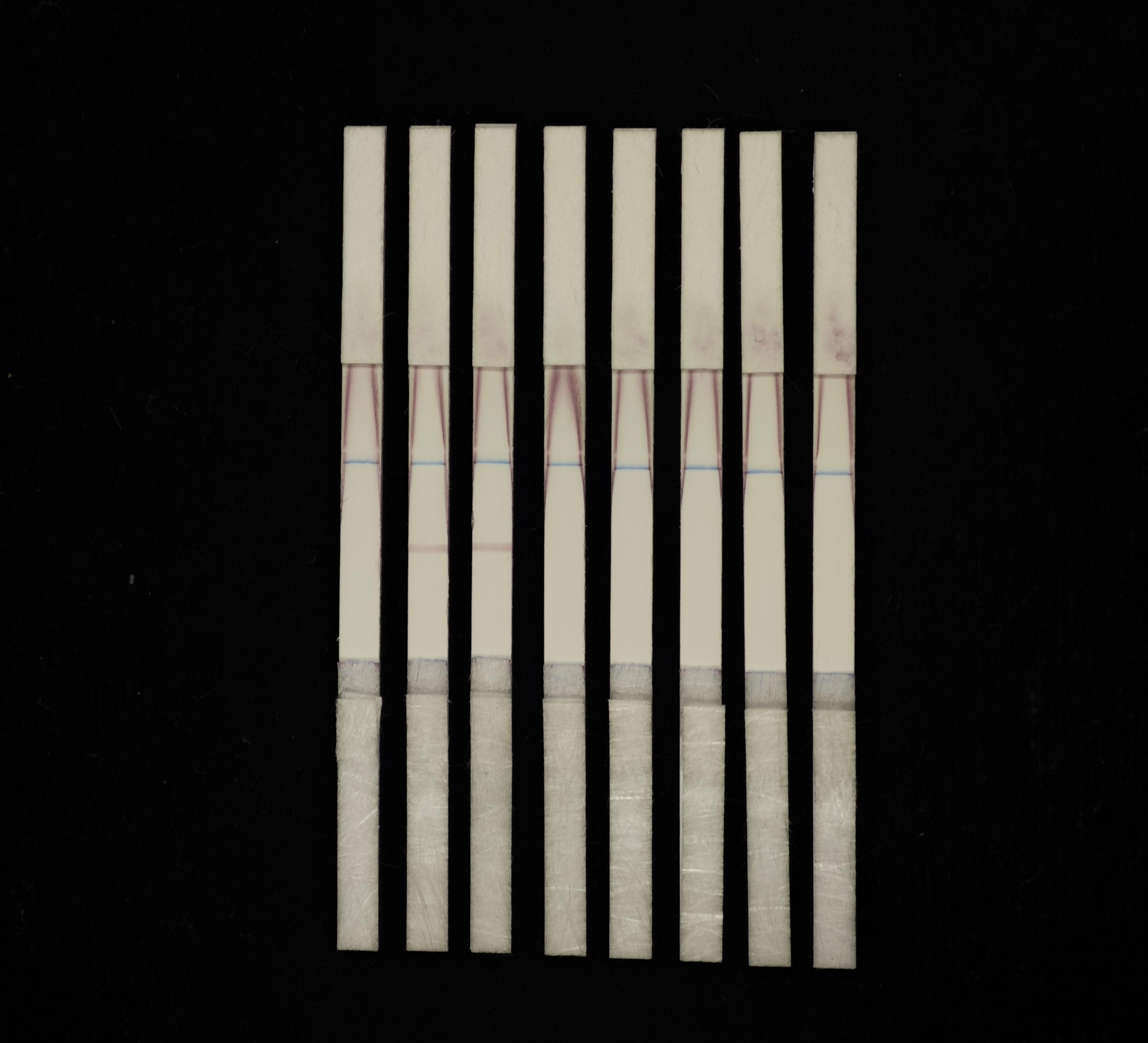

Supplement: Supplemental Information 4 [file peerj-14-21155-s004.zip › Uncropped figures/Sensitivity evaluation of the RAA-LFD assay-10min.jpg]

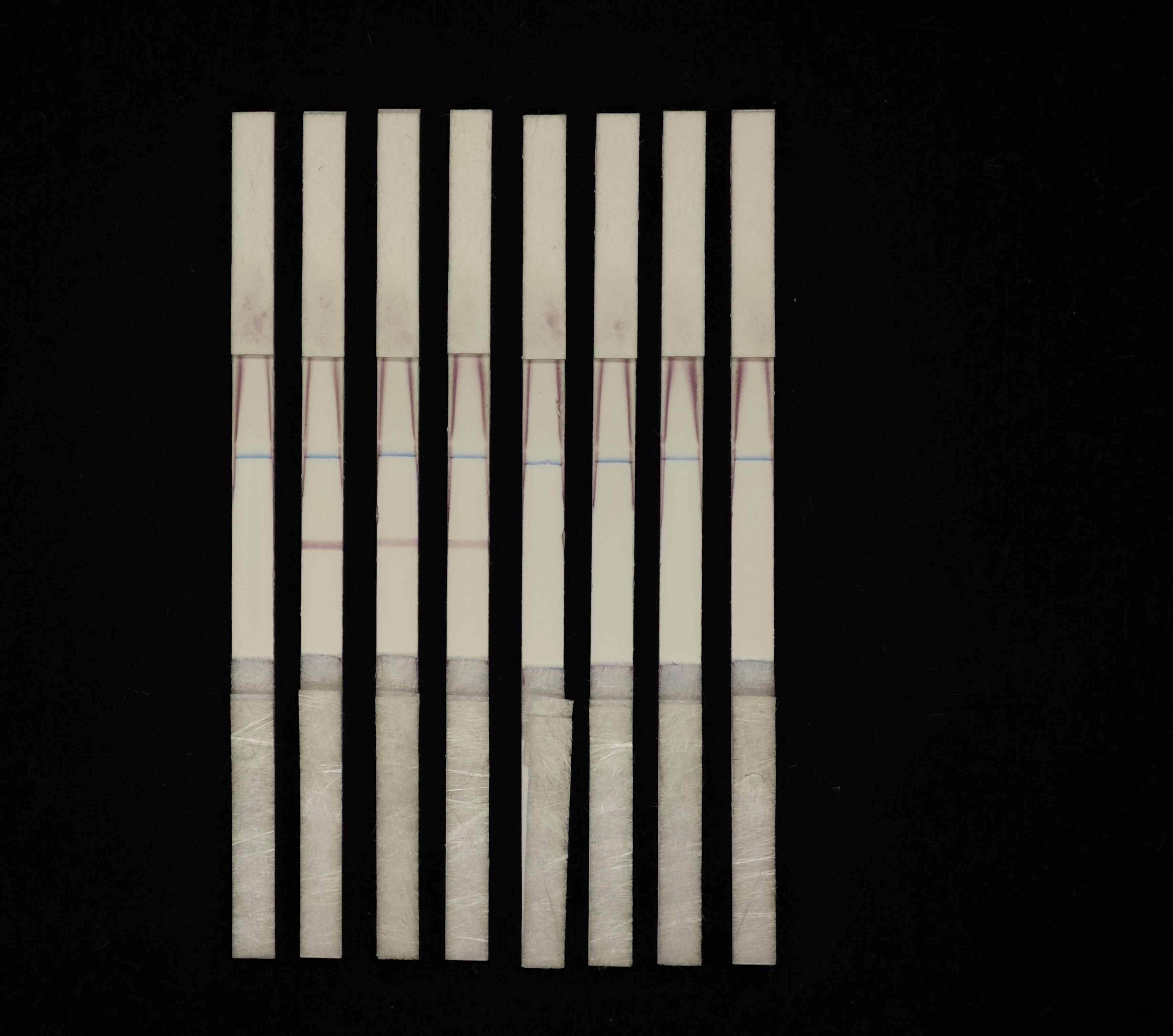

Supplement: Supplemental Information 4 [file peerj-14-21155-s004.zip › Uncropped figures/Sensitivity evaluation of the RAA-LFD assay-15min.jpg]

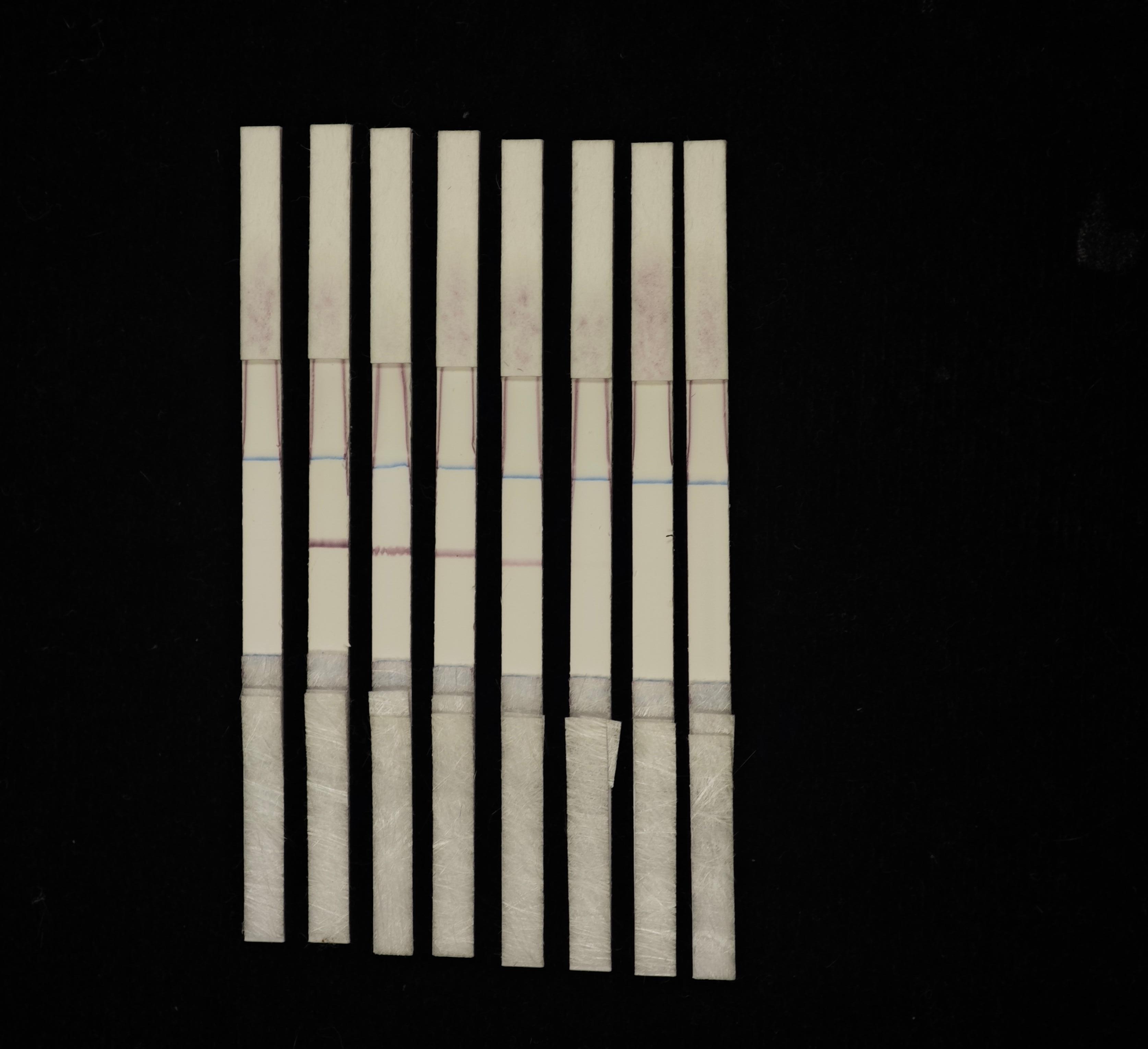

Supplement: Supplemental Information 4 [file peerj-14-21155-s004.zip › Uncropped figures/Sensitivity evaluation of the RAA-LFD assay-20min.jpg]

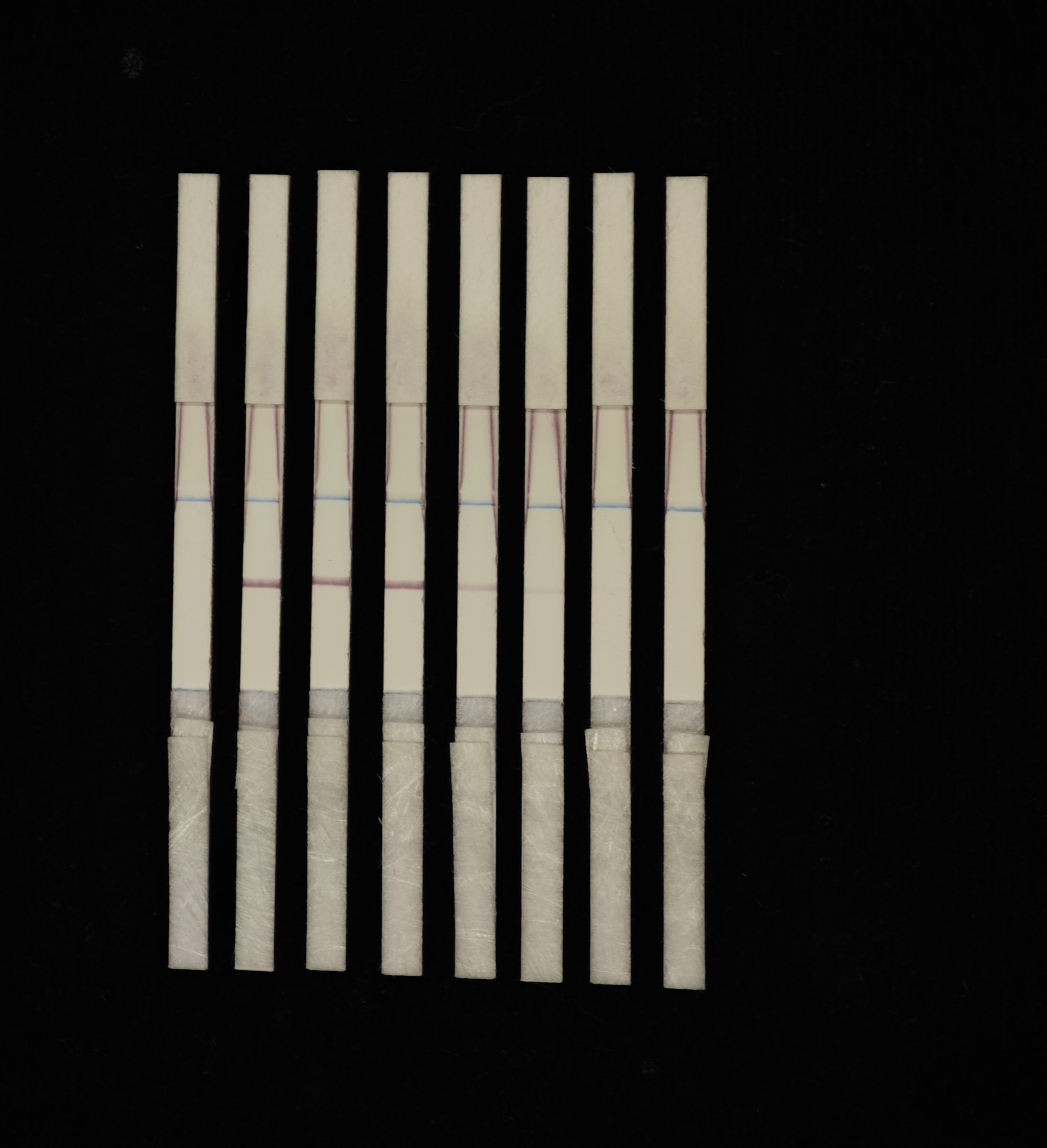

Supplement: Supplemental Information 4 [file peerj-14-21155-s004.zip › Uncropped figures/Sensitivity evaluation of the RAA-LFD assay-25min.jpg]

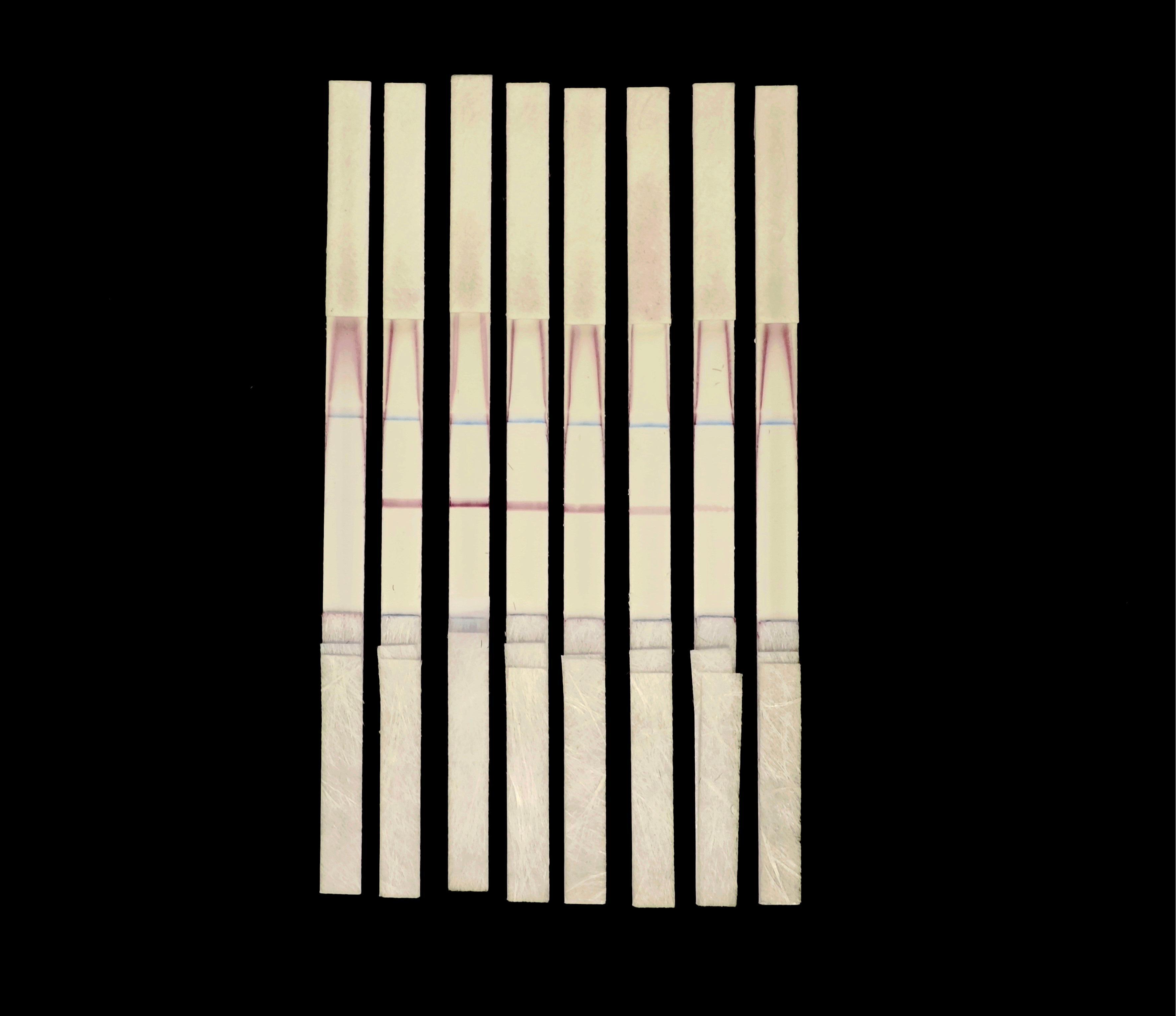

Supplement: Supplemental Information 4 [file peerj-14-21155-s004.zip › Uncropped figures/Sensitivity evaluation of the RAA-LFD assay-30min.jpg]

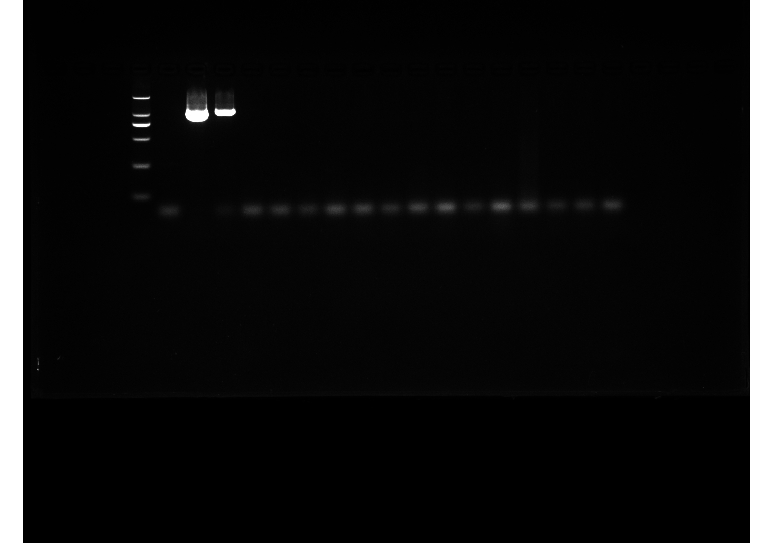

Supplement: Supplemental Information 4 [file peerj-14-21155-s004.zip › Uncropped figures/Specificity Evaluation of PCR Assay.jpg]

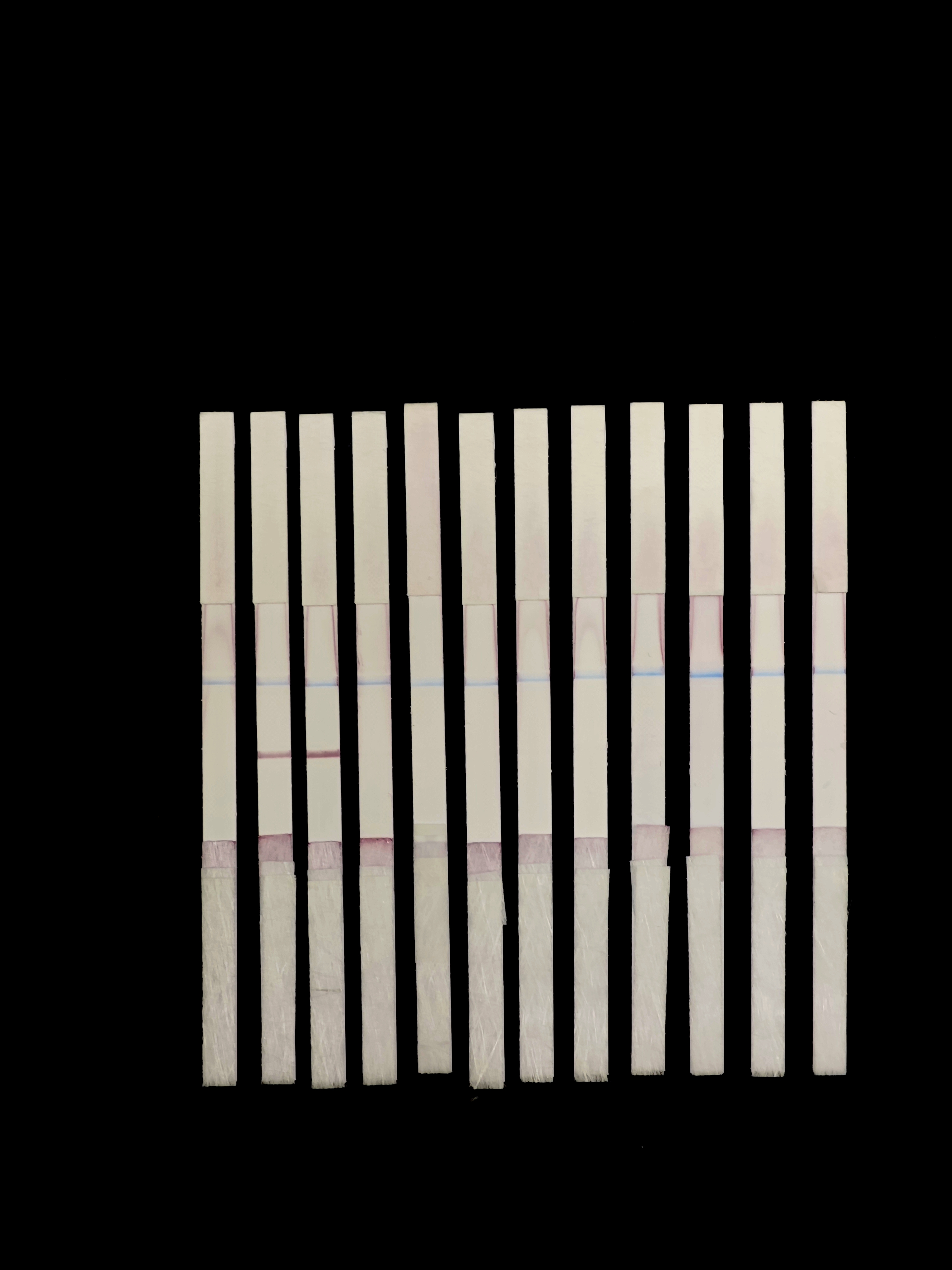

Supplement: Supplemental Information 4 [file peerj-14-21155-s004.zip › Uncropped figures/Specificity Evaluation of RAA Assay.jpg]
